# SARS-CoV fusion peptides induce membrane surface ordering and curvature

**DOI:** 10.1038/srep37131

**Published:** 2016-11-28

**Authors:** Luis G. M. Basso, Eduardo F. Vicente, Edson Crusca Jr., Eduardo M. Cilli, Antonio J. Costa-Filho

**Affiliations:** 1 Grupo de Biofísica Molecular Sérgio Mascarenhas, Instituto de Física de São Carlos, Universidade de São Paulo, Avenida Trabalhador São-carlense, 400, Centro, São Carlos, SP, Brazil; 2 Laboratório de Biofísica Molecular, Departamento de Física, Faculdade de Filosofia, Ciências e Letras de Ribeirão Preto, Universidade de São Paulo. Av. Bandeirantes, 3900, 14040-901, Ribeirão Preto, SP, Brazil; 3 Faculdade de Ciências e Engenharia, UNESP – Univ Estadual Paulista, Campus de Tupã. Rua Domingos da Costa Lopes, 780, 17602-496, Tupã, SP, Brazil; 4 Departamento de Bioquímica e Tecnologia Química, Instituto de Química, UNESP – Univ Estadual Paulista. Rua Prof. Franscisco Degni, 55, 14800-900, Araraquara, SP, Brazil

## Abstract

Viral membrane fusion is an orchestrated process triggered by membrane-anchored viral fusion glycoproteins. The S2 subunit of the spike glycoprotein from severe acute respiratory syndrome (SARS) coronavirus (CoV) contains internal domains called fusion peptides (FP) that play essential roles in virus entry. Although membrane fusion has been broadly studied, there are still major gaps in the molecular details of lipid rearrangements in the bilayer during fusion peptide-membrane interactions. Here we employed differential scanning calorimetry (DSC) and electron spin resonance (ESR) to gather information on the membrane fusion mechanism promoted by two putative SARS FPs. DSC data showed the peptides strongly perturb the structural integrity of anionic vesicles and support the hypothesis that the peptides generate opposing curvature stresses on phosphatidylethanolamine membranes. ESR showed that both FPs increase lipid packing and head group ordering as well as reduce the intramembrane water content for anionic membranes. Therefore, bending moment in the bilayer could be generated, promoting negative curvature. The significance of the ordering effect, membrane dehydration, changes in the curvature properties and the possible role of negatively charged phospholipids in helping to overcome the high kinetic barrier involved in the different stages of the SARS-CoV-mediated membrane fusion are discussed.

Severe Acute Respiratory Syndrome (SARS) is a viral respiratory illness caused by the SARS coronavirus (SARS-CoV) that affected 8,098 people worldwide, provoking 774 deaths^1^. Similarly to other enveloped viruses, SARS-CoV enters cells through fusion of its viral membrane with a host cell membrane. This fusion process is mediated by the spike (S) glycoprotein, a 1,255-amino acid type I transmembrane protein^2^ that assembles into trimers on the virion surface to form the characteristic spike structure of the SARS-CoV. These spikes are essential for the infection of the host cell and are responsible for both binding to cellular receptors (via S1 subunit)^3^,^4^ and fusion of viral and target cell membranes (via S2 subunit)^5^ in pH-dependent (endocytic)^6^ or independent (nonendocytic) pathways^7^.

Although cleavage of the S protein into S1 and S2 subunits enhances cell-cell fusion^8^, it does not seem to be a necessary requirement for virion entry^9^. Interestingly, SARS-CoV S protein exhibits multiple cleavage sites (for a review see Belouzard *et al*.^10^) and, so far, no direct correlation between fusion competency and the position(s) of the cleavage site(s) has been found. Besides the heptad repeat regions (HR1 and HR2), which are responsible for the formation of the characteristic coiled-coil six-helix bundle (6-HB), a common post-fusion structural motif shared by all class I viral glycoproteins^11^, the S2 subunit also contains internal membranotropic domains that play important roles in the fusion mechanism^10^,^12^. These functional domains, which include the so-called fusion peptide (FP), are exposed for membrane interaction upon a specific triggering (e.g., receptor binding or low endosomal pH). To date, three membrane-active and potential FP candidates have been identified in between HR1 and the N-terminus of the SARS-CoV S2 subunit: two highly conserved sequences across the *Coronaviridae*, corresponding to residues 864–886 (immediately positioned N-terminally to HR1)^13^ and to residues 798–815 (immediately positioned C-terminally to a second, internal cleavage site S2’ at R797)^14^, and another less conserved region corresponding to a hydrophobic stretch located between residues 770 and 788 (near the S1/S2 boundary region at site R667)^15^. These putative FPs are thought to destabilize host cell membranes, driving the refolding of the S2 subunit into the post-fusion 6-HB configuration, one of the late steps in the viral membrane fusion process^16^.

Although membrane fusion promoted by class I viral glycoproteins, such as SARS-CoV Spike, human immunodeficiency virus (HIV) gp160 or influenza virus hemagglutinin (HA), has been broadly studied in recent years^16^,^17^,^18^,^19^, many aspects of the molecular mechanism behind the virus-host cell membrane fusion remain unknown, including conformational changes of the lipid bilayers during peptide-membrane interactions.

Elucidating the nature of protein-lipid interactions as well as the conformational properties of both the membranotropic segments of the viral fusion proteins and the lipids in cell membranes can help to dissect the major steps of the orchestrated membrane fusion mechanism promoted by those biological machines. However, structural and dynamics information at the molecular level of peptide-induced membrane fusion in the context of the whole Spike protein is difficult to obtain. Thus, synthetic peptides corresponding to the putative fusion peptides might be very useful in providing detailed information on the interaction of those segments with lipid model membranes not only because the peptides themselves support membrane fusion, but also because there is a direct correlation between the effects of mutations in the intact protein and in the peptide analogues for membrane fusion^19^,^20^,^21^.

In the present study, we investigated the effects of two putative fusion peptides from SARS-CoV S glycoprotein, corresponding to residues 770–788 (SARS_FP_) and 873–888 (SARS_IFP_)^13^,^15^,^22^,^23^, on the structural dynamics, physicochemical properties, and thermotropic phase behavior of lipid model membranes by differential scanning calorimetry (DSC), continuous wave (CW) and pulsed electron spin resonance (ESR) along with nonlinear least-squares (NLLS) spectral fitting^24^. We found that both peptides increase the lipid packing and decrease the water content inside the lipid bilayer only for membranes containing negatively charged lipids, as well as generating opposing curvature stresses on highly curved membranes containing non-bilayer-forming phospholipids. The significance of the ordering effect, membrane dehydration, changes in the curvature properties and the possible role of negatively charged phospholipids for the fusion mechanism mediated by the SARS-CoV S glycoprotein are discussed.

## Results

We are interested in investigating the extent of perturbation of model membranes caused by a functional concentration of the peptides, i.e, peptide concentration that is already known to promote fusion of model membranes. At this concentration, what are the changes in the structural dynamics, curvature and hydration of the lipids and in the thermodynamic parameters of the membranes that lead to membrane fusion? It has been shown that SARS_FP_ and SARS_IFP_ are able to induce membrane fusion only at high peptide-to-lipid molar ratio^15^,^22^. Thus, we chose a 20:1 lipid/peptide molar ratio for most of the experiments and compared the effects of the peptides on model membranes that present very different physicochemical properties.

### Effects of the peptides on the melting behavior of lipid vesicles

**Figure 1:**
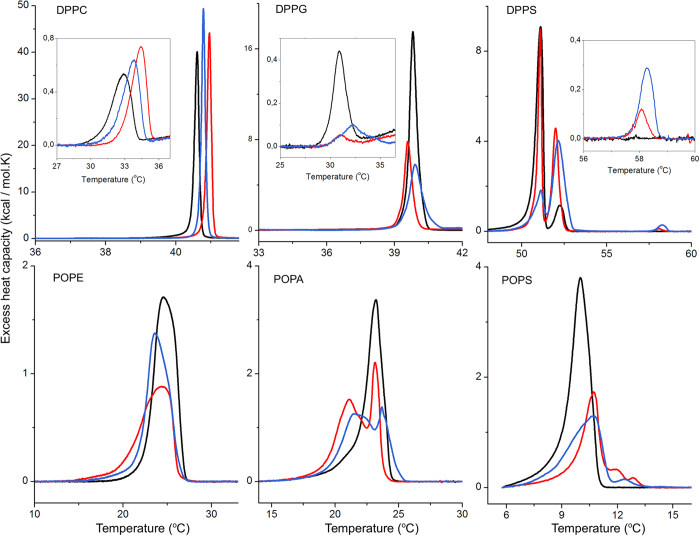
Perturbation of the thermodynamics of lipid phase transitions by the peptides. Representative thermograms illustrating the temperature dependence of the excess molar heat capacity of DPPC, DPPG, DPPS, POPE, POPA, and POPS vesicles without (black) and with incorporation of 5 mol% (20:1 lipid/peptide molar ratio) of SARS_FP_ (red) and SARS_IFP_ (blue). *Inset*: Effects of the peptides on the pretransition of DPPC and DPPG and also on the higher-temperature transition found in the peptide-containing DPPS vesicles. Buffer used was 20 mM potassium phosphate, pH 7.4.

To examine the effects of the putative SARS fusion peptides on the structural integrity of lipid model membranes, the thermotropic phase behavior of different multilamellar vesicles (MLV) in the absence and in the presence of 5 mol% of peptides were determined (Fig. 1). The thermodynamic parameters obtained from the analysis of the DSC curves are shown in Table 1. It is worth mentioning that the membrane-associated peptides remain folded in the liquid crystalline phase of the phospholipids^22^,^23^.

Multilamellar vesicles of DPPC and DPPG exhibited two endothermic events in the temperature range studied. The low-enthalpic, broad pretransition arises from the conversion of the lamellar gel phase, L_β’_, to the ripple gel phase, P_β’_, and is observed at about 32.9 °C for DPPC and at 30.9 °C for DPPG (Table 1). On the other hand, the more energetic and more cooperative (narrow) main phase transition arises from the conversion of P_β’_ to the liquid-crystalline phase, L_α_, and is centered at 40.6 °C for DPPC and at 39.8 °C for DPPG. Membranes composed of unsaturated lipids presented only a very asymmetric, low-enthalpic, and broad main phase transition at 23.2 °C for POPA, at 24.6 °C for POPE, and at ~10.0 °C for POPS. These results reasonably agree with the literature^25^, ^26^, ^27^, ^28^. The slight discrepancies observed in comparison with literature data are likely due to differences in lipid preparations, buffers, and ionic strength^29^,^30^,^31^. By contrast, the main phase transition of DPPS vesicles is split into two endothermic events, one centered at 51.1 °C and the other centered at 52.2 °C (Table 1). This two-peak feature has been attributed to different protonation states of the head group polar moiety^32^.

Binding of drugs, peptides, and proteins to membranes can promote structural and dynamic changes of lipid bilayers that significantly affect their thermotropic phase behavior^33^,^34^. Shifts of the melting transition temperature of the lipids, for instance, can be related to alterations of the entropy change between the gel and fluid states, whereas a broadening of the DSC thermogram may be the result of a decreased transition cooperativity (~1/ΔT_1/2_). As can be observed in Fig. 1, incorporation of 5 mol% of fusion peptides into the lipid bilayers did not strongly affect the main phase transition temperature of the liposomes. However, the enthalpy and entropy changes of the main phase transition were significantly altered, especially for membranes containing negatively charged lipids (Fig. 1 and Table 1).

SARS_FP_ and SARS_IFP_ only slightly perturbed zwitterionic DPPC liposomes. It was observed a small increase of both the melting temperature T_m_ (less than 1%) and the calorimetric enthalpy change of the transition ΔH (less than 2.5%), indicating a slight stabilization of the peptide-bound DPPC gel phase. The increased pretransition temperature of the peptide-containing DPPC vesicles also indicates structural changes in the DPPC polar head group^35^,^36^. On the other hand, more prominent effects on the enthalpy change and on the cooperativity of the transition of zwitterionic POPE vesicles were observed. SARS_FP_ decreased the transition ΔH by about 25%, whereas SARS_IFP_ decreased it by 16% (Table 1). POPE-SARS_FP_ interaction also markedly broadened the DSC endotherm (increase of ΔT_1/2_ by ~38%). This result indicates that SARS_FP_ intercalates within POPE bilayer and thus decreases the intermolecular cooperativity of the transition.

Overall, more significant effects on the thermodynamic (or DSC) parameters of the bilayer phase transitions were observed for membranes containing negatively charged lipids (Table 1): reduction of the calorimetric ΔH by about 51% (39%) for DPPG and by about 48% (43%) for POPS, for instance, after incorporation of SARS_FP_ (SARS_IFP_). Unlike the previous cases, endotherms containing multiple peaks appeared when the peptides were mixed with POPA vesicles (Fig. 1). In this case, the lower-temperature endothermic peak certainly arises from peptide-bound membranes, whereas the higher-temperature peak can be due to peptide-free POPA vesicles in the POPA/SARS_FP_ samples and due to a mixture of peptide-free and peptide-bound POPA vesicles in the POPA/SARS_IFP_ samples.

The two-component feature of DPPS main phase transition remained in the peptide-containing DPPS samples. Although the calorimetric enthalpy change of the whole transition decreased in the presence of the peptides, the area under the higher-temperature endotherm increased. The peptides also promoted opposing effects on the cooperativity of the two endotherms: while SARS_FP_ narrowed both transitions (thus increasing lipid cooperativity), SARS_IFP_ broadened the endotherms. This may be related to different locations and topology of the peptides in the membranes. Another interesting feature is the appearance of a third, low-enthalpic peak at around 58 °C in DPPS/peptide vesicles. This result indicates a different mechanism of PS-peptide interaction compared to the other negatively charged lipids. The low-enthalpy, higher-temperature peak (58 °C) was also observed in DMSO-treated peptide-containing DPPS liposomes, but was not observed in the acetonitrile-treated or DMSO-treated peptide-free DPPS vesicles. This result indicates that the new peak is not due to the binding of acetonitrile or DMSO from the peptide stock solutions to the DPPS MLVs (see Fig. S2 and Table S1 in supplementary information). Interestingly, a low-enthalpy peak also appeared in the peptide-bound POPS vesicles at a temperature slightly higher than the T_m_ of the peptide-free POPS vesicles (Fig. 1). Thus, this more stable and much less energetic endothermic peak may be due to specific PS-peptide interaction.

Taken together, these results suggest different mechanisms for the interaction of the peptides with zwitterionic and negatively charged phospholipids. Electrostatic interactions seem to play an important role for the SARS-CoV fusion peptide-membrane interactions, since both peptides are positively charged at pH 7.4: SARS_FP_, +2 e SARS_IFP_, +1. Moreover, since both peptides also significantly changed the thermotropic phase behavior of DPPG MLVs at high ionic strength (150 mM NaCl; reduction of ΔH by 34% for SARS_FP_ and by 52% for SARS_IFP_ – Table 1), hydrophobic interactions may also play an important role in peptide binding and penetration into membranes. Interestingly, the peptides promoted different effects on the thermograms of DPPG, DPPS, POPS, and POPA, suggesting that not only the charge but also the lipid packing (DPPS *vs.* POPS) and the lipid polar head group (PG, PS, or PA) may contribute to the energetics of peptide-membrane interactions.

### Membrane curvature-promoting properties

Since changes in membrane curvature have been associated with the potential mechanistic role of fusion peptides in inducing membrane fusion^37^,^38^, we tested the ability of the SARS-CoV fusion peptides to promote curvature strain on DiPoPE vesicles. Below the Lα-H_II_ phase transition temperature, T_H_, phosphatidylethanolamines, such as DiPoPE, spontaneously form lipid bilayers in the liquid-crystalline state, whereas above T_H_, they usually pack together in a highly curved hexagonally inverted structure with negative membrane curvature^39^,^40^. Stabilization of this concave curvature due to peptide binding, for instance, will favor the nonbilayer H_II_ phase, which will translate into a T_H_ reduction^20^. Conversely, peptides that induce positive membrane curvature will increase T_H_ of the DiPoPE/peptide samples and thus will stabilize the liquid-crystalline bilayer phase^41^.

**Figure 2:**
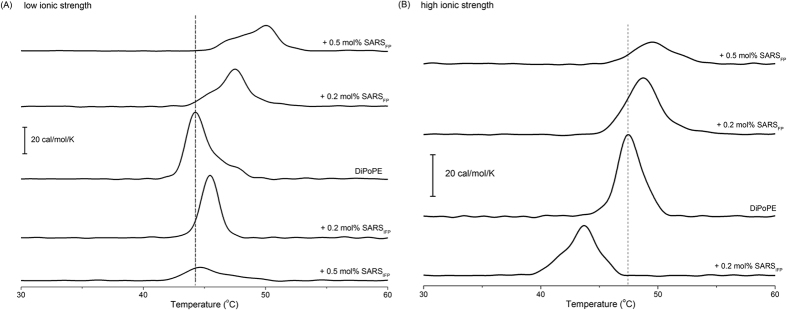
Curvature strain induced by SARS-CoV fusion peptides. Representative DSC traces illustrating the effects of the peptides on the liquid crystalline (Lα) to inverted hexagonal (H_II_) phase transition of DiPoPE multilamellar vesicles at the listed molar percentages and at low (**A**) and high (**B**) ionic strength. Buffer used was 20 mM sodium phosphate, pH 7.4, without or with 150 mM NaCl.

Figure 2 shows DSC traces of DiPoPE without and with 0.2 or 0.5 mol% of SARS_FP_ and SARS_IFP_ at both low (Fig. 2A) and high (Fig. 2B) ionic strength. The low-enthalpic Lα-H_II_ phase transition of DiPoPE was observed at 44.3 °C (ΔH_H_ = 136 cal/mol) and at 47.5 °C (ΔH_H_ = 107 cal/mol) at low and high ionic strength, respectively (Table 2), slightly higher than those observed in the literature^20^,^41^.

At low ionic strength, SARS_FP_ and SARS_IFP_ raised T_H_ and lowered the enthalpy change of the Lα-H_II_ transition. These results indicate that both peptides stabilize the lamellar liquid crystalline phase, Lα, of DiPoPE bilayers, with the most prominent changes in T_H_ observed for the more fusogenic SARS_FP_ peptide. On the other hand, SARS_FP_ and SARS_IFP_ induced opposing stresses on the intrinsic negative curvature of the DiPoPE H_II_ phase at high ionic strength. While SARS_FP_ increased T_H_ and stabilizes Lα phase, SARS_IFP_ lowered T_H_ and stabilized the inverted lipid H_II_ phase of DiPoPE/peptide vesicles. These findings imply that SARS_FP_ (SARS_IFP_) induced positive (negative) membrane curvature strain on DiPoPE vesicles at high ionic strength.

### Effects of the peptides on the structural dynamics of lipid vesicles

The local ordering and rotational dynamics of the lipid head group and acyl chains were investigated by CW ESR using the nitroxide-labeled lipids DPPTC, 5-PCSL, and 16-PCSL (see structure of the spin probes in Supplementary Fig. S1), which monitor different regions of the lipid bilayers. DPPTC reports on the lipid/water interface, whereas 5-PCSL and 16-PCSL monitor the hydrophobic core of the membranes at different depths of penetration^33^,^42^,^43^,^44^.

Despite the perturbation of the DPPC thermotropic parameters caused by the peptides, ESR spectra of the spin labels embedded into DPPC and DPPC/cholesterol 7/3 (mol/mol) MLVs at different temperatures did not show noticeable changes in the lipid structural dynamics of the peptide-containing vesicles as compared to the peptide-free lipid bilayers (see Supplementary Fig. S3). This means that the membrane-bound peptides do not significantly perturb the local structural dynamics of the head group or the hydrophobic core of the zwitterionic MLVs as investigated by CW ESR.

On the other hand, remarkable changes in the ESR spectra of the spin labels in liposomes containing negatively charged lipids, such as DPPG, DPPS, and POPA, were observed. The experimental and best-fit NLLS simulations are presented in Supplementary Figs S4 and S5 and the best-fit magnetic tensor components, rotational diffusion rates, and order parameters are summarized in Supplementary Tables S3–S6.

Before analyzing the changes in the lipid ordering and mobility induced by the peptides, it is worth mentioning a general feature observed for the order parameter S_0_ of DPPTC in the gel and the fluid phases of all lipid model membranes. We generally and consistently found a positive value for S_0_ in the gel phase of the lipids and a negative value in their fluid phase. For instance, we found S_0_ = −0.37 for DPPTC in DPPG at 45 °C (Table S3), S_0_ = −0.31 in DPPS at 60 °C (Table S4), and S_0_ ~ −0.29 in POPA at 25 °C and 37 °C (Table S5). The meaning of this negative value for S_0_ of DPPTC was extensively discussed by Ge and Freed for this same spin label in the fluid phase of DPPC dispersions (S_0_ = −0.32 at 50 °C) in terms of the analysis of the Cartesian components of the restoring potential U(Ω)^45^. In the gel phase, there is a molecular force field in the head group that tends to align the trimethyl ammonium (TMA) group of the DPPTC (Supplementary Fig. S1) perpendicular to the bilayer surface, i.e. parallel to the local director of the bilayer, giving rise to S_0_ > 0. However, in the fluid phase, that surface-orienting potential tends to align the TMA group parallel to the surface, giving rise to a negative order parameter^45^. By definition, S_0_ ≡ < (3cos^2^θ − 1)/2 > , hence S_0_ tends to −0.5 when θ tends to 90° and it is negative for 90° < θ < ~54.7°. Thus, if we compare the S_0_ values of DPPTC from our studies with that obtained by Ge and Freed, we might infer that the orientation of the TMA group in DPPC (S_0_ = −0.32; Ge and Freed work) and in DPPS (S_0_ = −0.31; our work) lies in between the one in DPPG (S_0_ = −0.37) and the other in POPA (S_0_ = −0.29). Those differences in the TMA orientation are likely due to distinct orienting forces that arise from the particular hydrogen-bonding network provided by the PG, PS, and PA bilayers and the dipolar interactions between the zwitterionic phosphoryl-Tempo-choline group of DPPTC and the surrounding negatively charged head groups. Additionally, irrespective of its sign, an increase of S_0_ (either by becoming more positive for S_0_ > 0 or less negative for S_0_ < 0) would lead to the same interpretation: a greater tendency of the preferential orienting axis of TMA to orient along the local director of the membrane and an increased restriction of the amplitude of its rotational motion.

**Figure 3:**
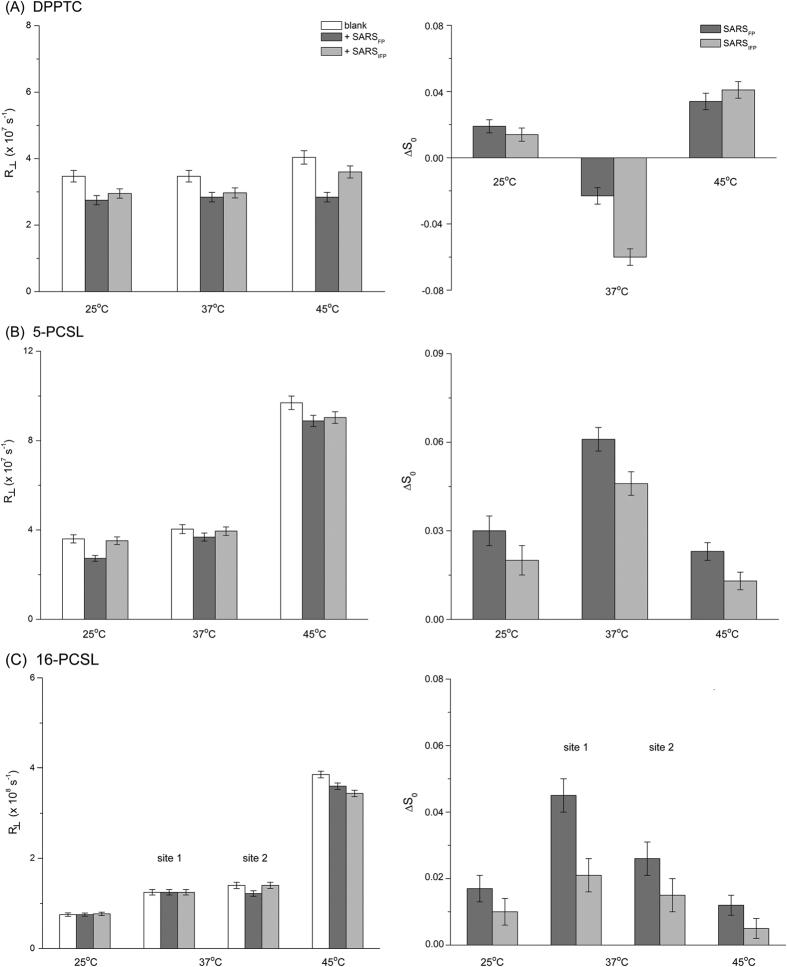
Changes in the ordering and mobility of different regions of DPPG bilayers in the gel and fluid phases. Plots of the rotational diffusion rate, R_⊥_ (left), and the variation of the order parameter, ΔS_0_ (right), of (**A**) DPPTC, (**B**) 5-PCSL, and (**C**) 16-PCSL incorporated in DPPG MLVs without (white) and with 5 mol% of SARS_FP_ (gray) and SARS_IFP_ (light gray) at 25 °C, 37 °C, and 45 °C. ΔS_0_ was calculated as the difference between the S_0_ obtained from the peptide-containing liposome with that of the peptide-free liposome. The 16-PCSL ESR spectra at 37 °C presented two components with different ordering and dynamics.

Figure 3 shows the plots of the rotational diffusion rates R_⊥_ and the changes in the order parameter S_0_ (ΔS_0_ = S_0_[peptide/lipid] − S_0_[lipid]) of DPPTC, 5-PCSL, and 16-PCSL in pure DPPG liposomes upon peptide addition at temperatures corresponding to the gel (25 °C), ripple gel (37 °C), and fluid (45 °C) phases of DPPG. As shown in Fig. 3A, both SARS_FP_ and SARS_IFP_ peptides significantly decreased the rotational diffusion of the head group at all temperatures, but only in the fluid phase the effect of both peptides are comparatively different from each other (R_⊥_ changes from 4.04 × 10^7^ s^−1^ to 2.84 and to 3.60 × 10^7^ s^−1^, for SARS_FP_ and SARS_IFP_, respectively – Table S3). This result indicates that DPPG-bound peptides remarkably restrict the mobility of the head group region. On the other hand, the order parameter of DPPTC increased by about 0.02 in the gel phase with peptide incorporation and by about 0.04 at 45 °C (Fig. 3A). Those changes correspond to the same level of alteration caused by the influenza hemagglutinin fusion peptide on the ordering of DPPTC in POPC (ΔS_0_ = 0.02) or in dimyristoylphosphatidylcholine (DMPC; ΔS_0_ = 0.05)^46^ or by the HIV gp41 fusion peptide on S_0_ in POPC/POPG 4/1 membranes (ΔS_0_ = 0.05) in the fluid phase^47^. In contrast, S_0_ decreased by about 0.02 and 0.06 at 37 °C. The reason for that is not quite clear, but a possible explanation would be the partitioning of DPPTC in the gel-like and fluid-like domains of DPPG in the ripple gel phase (DSC experiments; Fig. 1). Thus, the DPPTC ESR spectra at 37 °C may be regarded as a single averaged spectrum from which both gel-like and fluid-like populations are not resolved. Therefore, the effect of the peptides on R_⊥_ and S_0_ should be analyzed with caution in this case.

The ordering effect of the peptides on the DPPG head group region in the gel and fluid phases was also observed in the hydrophobic core of the bilayer at all temperatures, but with the most prominent changes in the ripple gel phase (Fig. 3B,C). The more fusogenic SARS_FP_ consistently increased the ordering of 5-PCSL and 16-PCSL more than SARS_IFP_. Particularly, since the 16-PCSL probe is more sensitive to molecular motions than DPPTC and 5-PCSL^43^,^48^, the structural dynamics of the gel-like and fluid-like lipid states could be resolved and characterized (Table S3). Although the peptides shifted the equilibrium between the two states towards the fluid-like one (the population of the fluid-like component increased from 10 to ~25%), the packing (S_0_) of the center of the bilayer was also increased for both lipid states. As for the mobility, the peptides reduced the rotational diffusion of 5- and 16-PCSL mainly in the fluid phase. Only SARS_FP_ was able to decrease R_⊥_ below T_m_, with the most significant change observed for 5-PCSL at 25 °C (R_⊥_ decreased from 3.60 × 10^7^ s^−1^ to 2.76 × 10^7^ s^−1^; Table S3).

**Figure 4:**
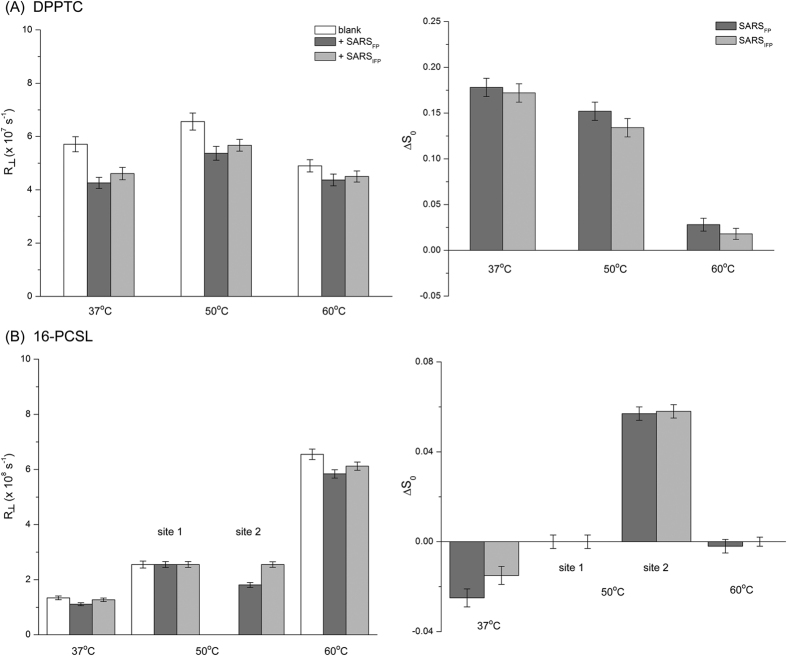
Changes in the ordering and mobility of different regions of DPPS bilayers in the gel and fluid phases. Plots of the R_⊥_ (left) and ΔS_0_ (right) of (**A**) DPPTC and (**B**) 16-PCSL incorporated in DPPS MLVs without (white) and with 5 mol% of SARS_FP_ (gray) and SARS_IFP_ (light gray) at 37 °C, 50 °C, and 60 °C. The 16-PCSL ESR spectra at 50 °C presented two components with different ordering and dynamics.

Figure 4 shows the R_⊥_ and ΔS_0_ values for DPPTC and 16-PCSL embedded in DPPS at temperatures corresponding to the gel (37 and 50 °C) and fluid (60 °C) phases of the membrane. The rotational mobility and order parameter of DPPTC were both affected by the peptides at all temperatures, but the most striking changes were observed in the very ordered DPPS gel phase (Fig. 4A). In the fluid phase, R_⊥_ decreased by 8% and 11% for SARS_IFP_ and SARS_FP_, respectively. At 37 °C, R_⊥_ decreased by 20–25%: from 5.71 × 10^7^ s^−1^ to 4.61 × 10^7^ s^−1^ for SARS_IFP_, and to 4.26 × 10^7^ s^−1^ for SARS_FP_ (Table S4). The most prominent change in the DPPS head group region induced by the peptides, based on our NLLS simulations, took place on the molecular alignment of the DPPTC TMA group. Firstly, the S_0_ at 37 °C found in our simulations was 0.26, much lower than that found in pure DPPG at the same temperature (*S*_0_ = 0.49; Table S3). Ge and Freed^45^ reported a value of 0.50 for DPPTC in DPPC dispersions in the gel phase (25 °C) and a similar value was found by Barroso *et al*.^42^ for a similar PC spin probe in an equimolar mixture of DPPC/DPPS (S_0_ = 0.52). This result indicates that the phosphoryl-Tempo-choline group of DPPTC is more loosely packed in the gel phase of the pure DPPS bilayer than in the other model membranes reported. Interestingly, peptide addition to pure DPPS remarkably increased the ordering of the head group in the gel phase: S_0_ jumped from 0.26 to about 0.43 for both peptides at 37 °C and similar changes were observed at 50 °C. In the fluid phase, though, just a slight variation was obtained (ΔS_0_ = 0.02–0.03) (Fig. 4A). Peptide binding to DPPS head group caused a large ordering effect, thus restricting the mobility of the lipids.

The spectra of 5-PCSL in the peptide-free DPPS obtained at temperatures below T_m_ presented very broad resonance lines (Supplementary Fig. S5B; 37 °C). Even in high ionic strength condition, a very broad lineshape in the ordered DPPS gel phase persisted (not shown). This line broadening effect is due to strong dipolar interactions that arise from possible cluster formation of the PC spin labels in the DPPS milieu. To test this hypothesis, we prepared DPPS/5-PCSL samples with either 0.25 mol% or 1.0 mol% of the spin label. At the higher probe concentration, even broader lines were obtained as compared with the ESR lineshape of 0.5 mol%, whereas narrower, but still broad, lines were found with 0.25 mol%. This result shows that 5-PCSL does not partition very well in the DPPS gel phase but does so in the fluid phase, as illustrated in Supplementary Fig. S5B (spectrum acquired at 60 °C). After unsuccessful attempts to fit the 5-PCSL spectra with the addition of a Heisenberg exchange coupling, we presented only the best-fit parameters from NLLS simulations of the spectra acquired at 60 °C (Supplementary Table S4). Both R_⊥_ and S_0_ were only slightly affected by the membrane-bound peptides at that temperature: R_⊥_ slightly decreased from 1.47 × 10^8^ s^−1^ to 1.34 × 10^8^ s^−1^ for SARS_FP_ and S_0_ increased from ~0.30 to ~0.31 or 0.32 for SARS_IFP_ and SARS_FP_, respectively (Table S4). Interestingly, binding of the peptides to DPPS promoted a better partition of 5-PCSL in the membrane. The structural organization of the DPPS/peptide membranes altered in such a way that the PC spin probe became miscible in the binary system.

As for the 16-PCSL in DPPS, no change in the ESR lineshape at both 25 °C and 37 °C upon peptide addition was observed (not shown), indicating no long range effect of the peptides on the center of the bilayer, despite the fact that they do bind and perturb the head group and the region around C_5_ of the lipid acyl chain. However, at 50 °C, SARS_FP_ and SARS_IFP_ promote the appearance of a second, disordered component in the ESR spectra (Supplementary Fig. S5B), not observed in the spectrum of the peptide-free DPPS/16-PCSL. This additional spin population (25% induced by SARS_FP_ and 12.5% by SARS_IFP_) presented characteristics of a fluid-like lipid state (S_0_ ~ 0.06 and rotational correlation time on the sub-nanosecond time scale; Table S4). In the fluid phase, the only parameter affected by peptide binding was the rotational mobility, which was diminished from 6.55 × 10^8^ s^−1^ to 6.12 or to 5.84 × 10^8^ s^−1^ for SARS_FP_ or SARS_FP_, respectively. Our results indicate the peptides bind to both gel and fluid phases of DPPS, but peptide insertion into the membrane seems to be activated either by temperature or ultimately by loosening of the hydrophobic packing of the highly ordered DPPS gel phase. As the temperature is increased, long range effects on R_⊥_ of the acyl chain were observed in the bilayer center.

**Figure 5:**
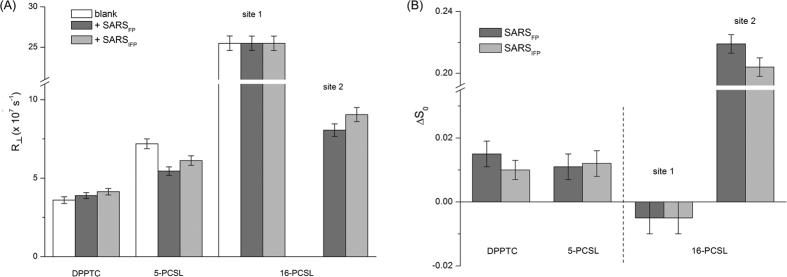
Changes in the ordering of different regions of POPA bilayers in the fluid phase. Plots of ΔS_0_ of (**A**) DPPTC, (**B**) 5-PCSL, and (**C**) 16-PCSL incorporated in POPA MLVs without (white) and with 5 mol% of SARS_FP_ (gray) and SARS_IFP_ (light gray) at 25 °C and 37 °C. The 16-PCSL ESR spectra at 25 °C presented two components with different ordering and dynamics.

We also studied the effect of the peptide binding on the structural dynamics of POPA MLVs in the fluid phase (25 °C and 37 °C). As shown in Table S5, SARS_FP_ and SARS_IFP_ only changed the ordering and R_⊥_ of the spin labels at 25 °C (Fig. 5). At 37 °C, the ESR spectra from peptide-free and peptide-bound vesicles are almost the same. Differently from the other model membranes, peptide binding to POPA head group causes an increase of the mobility: R_⊥_ changed from 3.60 to 4.14 × 10^7^ s^−1^ for SARS_IFP_ and to 3.89 × 10^7^ s^−1^ for SARS_FP_ (Fig. 5A). This result is probably related to the lack of a bulky head group of POPA (the surface area per lipid of PA head group is smaller than that of PG or PS), which may facilitate the rotational diffusion of the phosphoryl-Tempo-choline group of DPPTC, despite the small decrease in the amplitude of the TMA molecular motion (S_0_ increased by about 0.01 to 0.02; Fig. 5B). On the other hand, the best-fit NLLS simulations of 5-PCSL spectra showed a prominent effect on R_⊥_ rather than S_0_: while the rotational mobility diminished 15% for SARS_IFP_, it decreased 24% for SARS_FP_, whereas ΔS_0_ was only about 0.01 (Supplementary Table S5). As for the 16-PCSL, we found a very high value for S_2_ (−0.30) in the peptide-free POPA liposomes, indicating a large deviation from cylindrical symmetry of the molecular alignment of the end-chain label to the local director of the membrane. To gain further insights on the molecular orientation of the end-chain label we derived the orienting potentials along the Cartesian directions from the potential coefficients obtained by the best fits of the NLLS simulations (section SI2): u_x_ ≡ U(90°, 0°)/kT = 1.81, u_y_ ≡ U(90°, 90°)/kT = −0.83, and u_z_ ≡ U(0°, 0°)/kT = −0.98. This result indicates a strong preference for the rotational diffusion axes z_R_ (parallel to the 2pz orbital of the nitrogen) and y_R_ of the nitroxide moiety to align along the normal to the bilayer, while x_R_, which is parallel to the N‒O bond, is prevented to align along the membrane local director. Therefore, the unsaturation of the oleyl acyl chain of POPA would allow for a dynamic bending of the end chain of the spin label that favors much more the alignment of both z_R_ and y_R_ axes to the bilayer vector than x_R_, the direction of the N‒O bond. This same effect was observed by Ge *et al*. with 16-PCSL in POPC/POPS model membranes^49^.

The most striking effect of both peptides on the 16-PCSL lineshape was the appearance of a second, more ordered and highly-populated (50‒60%) component (Supplementary Fig. S5C) with almost no change in ordering and dynamics of the other component. This second site is characterized by the following parameters: S_0_ = 0.22, S_2_ = 0, and R_⊥_ ~ 8 × 10^7^ s^−1^, for SARS_FP_; and S_0_ = 0.20, S_2_ = 0, and R_⊥_ ~ 9 × 10^7^ s^−1^, for SARS_IFP_. These parameters are significantly different from those corresponding to the single component in the peptide-free sample: S_0_ = 0.15, S_2_ = −0.30, and R_⊥_ ~ 2.6 × 10^8^ s^−1^ (Supplementary Table S5). Since the fluid-state lipids (37 °C) present lower order (S_0_ = 0.10) and high degree of molecular misalignment (S_0_ = −0.27) with R_⊥_ ranging from 2.5 to 3.0 × 10^8^ s^−1^ (Supplementary Table S4), these results allow us to infer that this second component does not present the same properties of fluid-state lipids but rather peptide-bound lipids. That is, peptide binding to POPA bilayers gives rise to peptide-enriched and peptide-free domains, where the latter is barely affected by the peptides. affected by the peptides. These results are in good agreement with our DSC experiments, in which, as discussed earlier, thermograms with multiple peaks arise from peptide-associated and peptide-free POPA vesicles.

**Figure 6:**
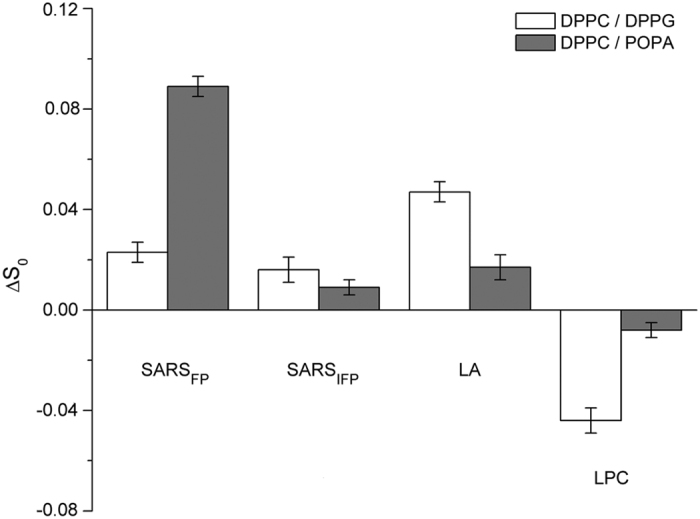
Effect of membrane fusion promoter and inhibitor on the membrane surface ordering of lipid bilayers. Variation of the order parameter S_0_ of DPPTC in equimolar mixtures of DPPC/DPPG (white) and DPPC/POPA (gray) multilamellar vesicles upon incorporation of 5 mol% of SARS_FP_ and SARS_IFP_ or 10 mol% of LA and LPC relative to the pure bilayer.

Additionally, it is important to investigate the effects of membrane fusion promoters and inhibitors on the bilayer properties in an attempt to identify the changes in the physicochemical parameters of the membrane that might be relevant for membrane fusion. Generally speaking, insertion of inverted cone-shaped molecules such as the lysophosphatidylcholine 1-palmitoyl-2-hydroxyl-PC (LPC) into the outer monolayer would prevent membrane fusion supposedly by promoting positive membrane curvature^50^,^51^. Conversely, insertion of cone-shaped molecules such as phosphatidylethanolamine, arachidonic acid (AA), and linoleic acid (LA) into the outer monolayer would facilitate membrane fusion presumably by inducing negative curvature^52^. Using ESR, Ge and Freed have found opposing effects of LPC and AA on the order parameter of a headgroup spin label embedded in DMPC model membranes^46^. In their work, an ordering (disordering) effect was observed for the fusion promoter (inhibitor) AA (LPC), which may help to induce (prevent) membrane fusion. Since the putative fusion peptides from SARS-CoV S protein promote substantial membrane fusion only in the presence of negatively charged lipids, it is important to verify whether that correlation is also valid for negatively charged lipid-containing vesicles. To do so, we prepared equimolar mixtures of DPPC/DPPG and DPPC/POPA membranes and studied the effects of SARS_FP_, SARS_IFP_, LA, and LPC on the order parameter S_0_ of DPPTC at 37 °C. The best-fit parameters of the ESR spectra shown in Fig. S6 are summarized in Table S6 in supplementary information. Figure 6 shows that both fusion peptides and LA caused an ordering effect on the head group of both model membranes. In contrast, LPC promoted a disordering effect of DPPTC. These results are thus in accordance with those found by Ge and Freed for the zwitterionic DMPC^46^ and indicate that SARS_FP_ and SARS_IFP_ might facilitate membrane fusion similarly to how the fusion peptide from influenza hemagglutinin does.

### Dehydration of POPC/POPG membranes

Since the ordering effect of the lipid head group has been attributed to bilayer dehydration^53^, we used ESEEM spectroscopy to find out whether the peptide-induced ordering of anionic vesicles is possibly related to membrane dehydration, as suggested as a general fusion mechanism of other class I fusion peptides^46^,^47^. ESEEM has been successfully applied to examine the penetration depth profile of deuterium-substituted molecules, such as water, glycerol, and sugar inside lipid bilayers^54^,^55^,^56^,^57^ as well as to investigate the water permeation at protein/peptide-lipid interface of membrane-interacting peptides and membrane proteins^58^,^59^,^60^. Here we used a D_2_O-containing buffer to probe the deuterium environment surrounding the spin labels DOPTC, 5-PCSL, and 16-PCSL (Supplementary Fig. S1) embedded in POPC/POPG 7/3 (mol/mol) MLVs. The different spin labels allowed investigating the changes promoted by the peptides in the water content from the lipid/water interface down to the hydrophobic core of the bilayers. Changes in the modulation depth of the time-domain ESEEM spectra or in the deuterium spectral density yields information about D_2_O molecules situated near the nitroxide within a distance range of up to 1.0 nm, which is the known spatial reach of the method^56^.

**Figure 7:**
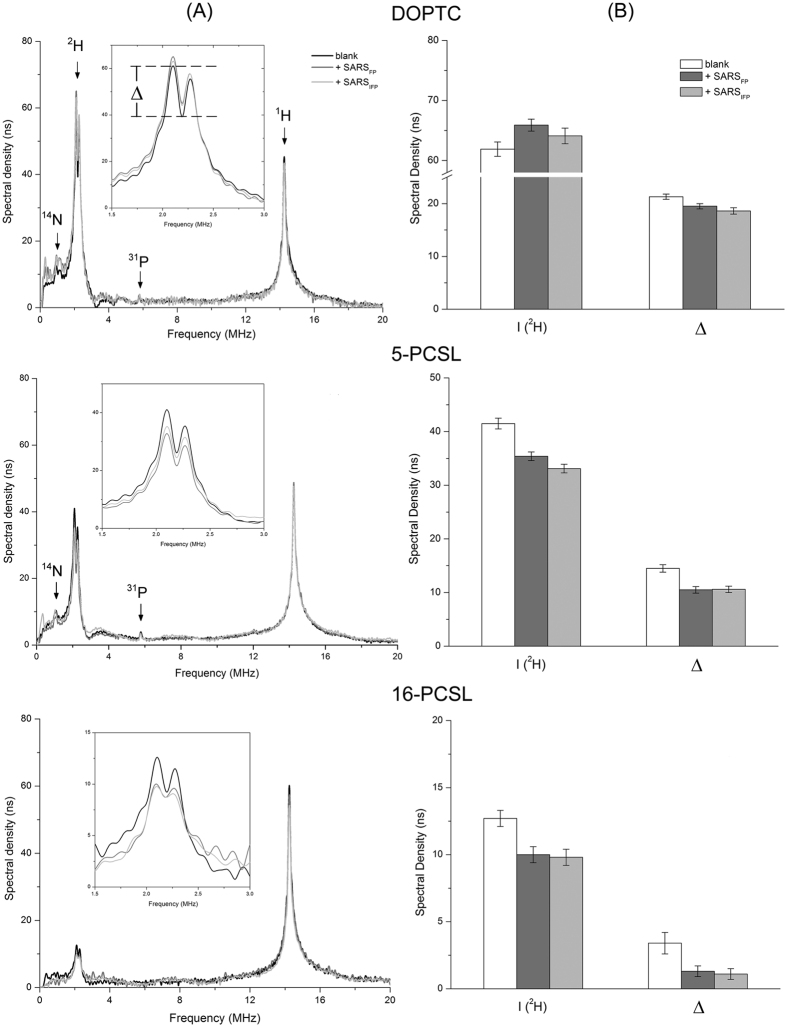
Membrane dehydration induced by the SARS fusion peptides. (**A**) Frequency-domain ESEEM spectra of DOPTC, 5-PCSL, and 16-PCSL embedded in peptide-free and peptide-bound POPC/POPG 7/3 (mol/mol) membranes. Insert: amplification of the low-frequency region corresponding to the deuterium signal. The intensity of the quadrupole interaction, Δ, defined as illustrated, is related to free D_2_O content between 0.5 to 1.0 nm from the nitroxide. (**B**) Corresponding spectral densities at 2.09 MHz, I(^2^H), and quadrupole doublet intensities, Δ, of the signals shown in (**A**).

Figure 7A shows the spectral density of the spin-labeled lipids in the peptide-free and peptide-containing lipid vesicles (the normalized time-domain stimulated echo signals are given in the Supplementary Fig. S7A). All spectra are dominated by two signals, one centered at 2.2 MHz, arising from the hyperfine interaction of the nitroxide with surrounding deuterium nuclei (^2^H-Larmor frequency at 344 mT), and the other centered at 14.25 MHz, which arises from matrix protons near the spin label. Since lipids, peptides and possibly residual H_2_O contribute to the spectral density at 14.25 MHz, the ^1^H signal was not further analyzed. Two additional weak peaks were observed in the FT-spectra of DOPTC and 5-PCSL: one at ~1.03 MHz, which is assigned to ^14^N^57^, and the other at ~5.77 MHz, which corresponds to the Larmor frequency of ^31^P (Fig. 7A)^59^. Those nuclei belong to the choline group of POPC (^14^N) and to the phosphate group (^31^P) of the lipids. Both peaks are absent in the 16-PCSL ESEEM spectra because the distance between the spin probe moiety in the bilayer midplane and the head group^61^ is beyond the limit of the technique.

The deuterium peak is composed of two spectral components: a low-intensity broad signal that arises from a direct deuterium bond between D_2_O and the nitroxide radical; and a narrow doublet of intensity I(^2^H) and amplitude Δ (Fig. 7A), which arises from the quadrupole interaction between the electron spin and non-^2^H-bonded water molecules inside the bilayer^54^. Both Δ and I(^2^H) have been found to depend linearly and nonlinearly, respectively, on the concentration of free water molecules at a distance of 0.5 to 1.0 nm^56^.

The parameters I(^2^H), measured at 2.09 MHz, and Δ for all lipid spin labels are shown in Fig. 7B. As expected, the deuterium spectral density decreases from the bilayer/water interface toward the membrane center. Particularly, I(^2^H) is reduced from (62 ± 1) ns in the water/lipid interface to (42 ± 1) ns around 5^th^ carbon position and to (12.7 ± 0.6) ns near the bilayer midplane in the peptide-free vesicles, indicating reduced water content in the hydrophobic core as compared to the head group region. Those I(^2^H) values are somewhat higher than previously reported spectral densities for POPC/POPG 4/1 (mol/mol) bilayers^59^ but are in the same order of magnitude of those for DPPC^54^,^62^. The discrepancies are most likely due to different freezing protocols, sample preparation, and experimental conditions such as the choice of τ, the first interpulse delay, and the frequency chosen to measure the intensity of the spectral density^55^.

SARS_FP_ and SARS_IFP_ decreased both I(^2^H) and Δ for the spin labels that monitor the hydrophobic region of the bilayer (Fig. 7B). This finding implies that both peptides interact with POPC/POPG membranes and displace free water molecules from their hydrophobic core. Theoretical calculations from Milov *et al*. indicated that nitroxide-deuterons interactions spread to around 1 nm^56^. Therefore, the spin label at C_5_ position is able to monitor water density around C_5_ up to the level of the carbonyl-glycerol and phosphocholine regions (distant about 1.0 nm from C_5_)^61^,^63^. Thus, the contribution from bulk water molecules to the spectral density of 5-PCSL is negligible^54^,^56^. The significant 27% decrease of Δ for 5-PCSL in the peptide-containing membranes, relative to the peptide-free vesicles (Supplementary Table S7, Fig. 7B), allows us to infer that the intramembrane water in the outer membrane region is dramatically reduced upon peptide incorporation. The same reasoning holds true for the 16-PCSL.

As for the head group spin label DOPTC, we found that the peptides surprisingly promoted opposing effects on I(^2^H) and Δ: while they slightly raised the magnitude of the D_2_O signal, Δ values were reduced (Fig. 7B). The source of this opposing effect remains unclear to us, but it may be of some interest to speculate on that. Considering the electron density profile of PC lipids^61^,^63^, we may infer that the nitroxide radical of the DOPTC phosphoryl-Tempo-choline group is in direct contact with both membrane surface and bulk water molecules. Changes in the orientation of the phosphoryl-Tempo-choline must therefore locally disturb the water density around the spin label. Incorporation of cholesterol into PC membranes, for instance, decreases the ordering of the lipid head group, i.e., makes it less aligned along the bilayer director^64^. This effect allows for water molecules to move from the bulk into the membrane (down to ~C_7_), thus increasing both I(^2^H) and Δ (Fig. S7B and Table S7 in supplementary information)^54^. On the other hand, if the phosphoryl-Tempo-choline group becomes more aligned along the bilayer normal, i.e. the S_0_ of DOPTC increases, membrane-surface dehydration takes place^53^. In this situation, the concentration of membrane-surface water molecules is reduced in the ‘membrane side’. In contrast, realignment of the head group dipole makes the nitroxide radical become more exposed to interact with bulk water molecules in the ‘solvent side’. Milov *et al*. have theoretically shown that the nonlinear dependence of the I(^2^H) on water concentration is due to the formation of nitroxide-water complexes^56^. Therefore, if the water distribution around the nitroxide is locally perturbed in such a way that it allows for formation of nitroxide-water complexes (i.e., disturbances take place closer than 0.5 nm), it must affect mainly the I(^2^H), but not Δ^56^. That is, reorientation of phosphoryl-Tempo-choline due to peptide-lipid interactions may be such that it allows formation of nitroxide-water complexes, affecting mainly I(^2^H). This may correspond to the source of the increased I(^2^H). The contribution to Δ stems mainly from water molecules belonging to the second hydration shell (>0.4 nm) up to ~1 nm. From the ‘solvent side’, the second water coordination sphere for the water-exposed nitroxide radical should not change, thus the major contribution to Δ of DOPTC may arise from free water molecules located in the ‘membrane side’. The reduced level of the head group hydration accounts therefore for the decrease of Δ values. However, further detailed studies are needed to investigate this hypothesis.

## Discussion

Viral membrane fusion is a concerted mechanism that involves remarkable protein and lipid conformational changes. The molecular mechanism by which viral fusion proteins catalyze the membrane fusion reaction and the molecular details of lipid rearrangements in the lipid bilayer are not fully understood yet, although the current model has been constantly revisited and refined^65^,^66^,^67^,^68^,^69^,^70^. Crystal structures of pre- and post-fusion states of class I viral fusion proteins along with NMR structures of fusion peptides in membrane mimetics have provided a mechanistic view of the membrane fusion process. In this process, different protein segments act in an orchestrated way to achieve the complex and kinetically unfavorable task of bringing together and fusing two lipid bilayers^16^,^18^. However, there are still major gaps in the molecular details involved in the fusion process. They include: the sequential order and kinetics of the events, changes in the structure, dynamics, and physicochemical properties of different protein domains and lipid bilayers during the whole membrane fusion mechanism, free energies of the corresponding pre-, post- (fusion pore) and intermediate (hemifusion) fusion states as well as the modulation of all those parameters by lipid composition.

SARS-CoV S2 subunit possesses various membranotropic segments, i.e., relatively short hydrophobic domains that are able to bind to and perturb membranes. These segments can act independently from each other and may help to stabilize the contact between viral and cell membranes^14^,^71^,^72^,^73^,^74^. However, monitoring the structural rearrangements of different segments of the intact S2 subunit as well as the dynamics of their interaction with viral and cell membranes on a molecular level constitute a challenging task. The use of synthetic peptides and phospholipids has provided important thermodynamic, structural, biochemical, and functional details of peptide-membrane interactions from both peptide and lipid perspectives, which makes them a good platform to study membrane fusion^20^,^70^,^75^.

Our results indicate that both SARS_FP_ and SARS_IFP_ significantly perturb the thermotropic phase behavior as well as the molecular ordering and phospholipid rotational mobility of model membranes containing negatively charged lipids, but only cause moderate effects on zwitterionic membranes. These findings are in agreement with previously reported studies that indicated greater disturbance and higher affinity of both peptides for model membranes containing anionic rather than zwitterionic phospholipids^22^,^23^. Even though the peptides do not partition well into zwitterionic membranes, they are able to perturb the thermodynamic parameters of the zwitterionic DPPC and POPE phase transitions as well as to change the membrane curvature properties of DiPoPE. On the other hand, our CW ESR experiments showed that the peptides do not affect the structural dynamics of zwitterionic lipid bilayers, but they do promote an ordering effect on the lipid head group and on the acyl chains of negatively charged membranes. This latter effect seems to be correlated to membrane dehydration, as shown by our ESEEM experiments. Ordering and dehydration effects have also been observed for other class I viral fusion peptides such as those from HIV-I gp41 and influenza HA glycoproteins^46^,^47^. The relevance of membrane-curvature induction and lipid dehydration for the viral membrane fusion of the SARS-CoV fusion peptides will be discussed below.

### Peptides induce bending moment and membrane dehydration of anionic membranes

Lipid molecules in bilayers experience a restoring torque from neighboring lipids that tends to reorient the lipid chain and/or the head group along the normal to the bilayer. The order parameter S_0_, which is associated with the restoring torque through the orienting potential U(Ω), is a measure of the extent of that molecular alignment. Generally, the higher the S_0_ the lower is the angular amplitude of the wobbling motion of the spin probe, i.e. the more aligned along the local director is the lipid segment to which the probe is attached. A better alignment can enhance the molecular interactions between lipid molecules in the bilayer. Therefore, S_0_ directly reports on the lipid packing density. In particular, slight changes in S_0_ of the lipid head group would have a greater impact on the structural organization of the membrane than changes in the ordering of the acyl chain^46^. This is primarily due to the strong hydrogen-bonding network in the head group region (~2.4 to 9.5 kcal/mol)^76^. Thus, although the molecular structure of DPPTC head group is different from those of the PG, PS, and PA head groups, reorientation of DPPTC due to conformational changes in the polar region might be associated with changes in the hydrogen-bonding network^53^. That is, the more ordered the DPPTC, the more condensed is the bilayer, which leads to changes in the ionic interactions between the head groups and between the head groups and surface-bound water molecules. By contrast, the van der Waals interactions between the lipid acyl chains in the hydrophobic core of the membrane are much weaker, i.e. the strength of the interaction is about 0.25 kcal/mol, which is even smaller than the thermal energy at 298 K (k_B_T ~ 0.59 kcal/mol)^76^. Nevertheless, enhancement of lipid-lipid interactions due to condensation of the hydrophobic core leads to an increased chain-packing energy.

In our studies, the peptides were added into preformed vesicle solutions. Therefore, the changes observed here are primarily due to the interaction of the peptides with the outer leaflet of the bilayers. SARS_FP_ and SARS_IFP_ increase the S_0_ of 5-PCSL and 16-PCSL in DPPG and POPA membranes, making the nonpolar core of the outer leaflet more solid-like. Furthermore, both peptides increase the ordering of head group spin label DPPTC in pure DPPG, DPPS, and POPA as well as in DPPG- and POPA-containing membranes, but not in the zwitterionic DPPC and DPPC/Chol. Therefore, an increase of both the head group and acyl chain packing densities of the outer leaflet is the major effect of the peptides on the negatively charged phospholipid membranes investigated in this work, which is in agreement with previous studies^22^,^23^. Interestingly, SARS_FP_ shows higher membrane fusion activity in pure PG or in PS- or phosphatidylinositol (PI)-containing membranes, but not in zwitterionic ones^22^. Thus, ordering of the lipid head group seems to be an important structural change in the bilayer necessary to induce membrane fusion^46^,^47^. Indeed, we found the same ordering effect on the head group of membranes containing negatively charged lipids for the membrane fusion promoter LA and an opposite effect for LPC, a known fusion inhibitor.

This ordering or condensation effect on the outer monolayer leads to a shrinkage of its surface area that compresses the inner leaflet. Because of the mechanical coupling between the two leaflets in a lipid bilayer vesicle, the inner layer counteracts the compressive force exerted by the outer monolayer and therefore creates a nonuniform tension in the two leaflets, which redistributes the stress profile across the bilayer. As a result, a uniform membrane bending moment toward the condensed leaflet is induced, which ultimately generates negative (positive) membrane curvature if the outer (inner) monolayer is condensed^77^. Due to the properties of the stress profile across the bilayer and to the nature of the molecular interactions in the head group and acyl chain regions, the largest contribution to the bending moment arises from the ordering of the head group region^46^. Thus, the increase of S_0_ of DPPTC in the outer leaflet of negatively charged lipid membranes promoted by the SARS-CoV fusion peptides could potentially induce negative membrane curvature (please see section SI3 of supplementary information for further details). This negative curvature effect induced by bending moment has also been proposed as the putative membrane fusion mechanism of other class I fusion peptides such as those from influenza HA or HIV-1 gp41^46^,^47^.

Another important aspect to consider is the inverse correlation between head group ordering and hydration of lipid bilayers^53^. If an increased head group packing density leads to membrane dehydration, this effect may help to overcome the high hydration repulsive energy that arises from membrane surface-bound water molecules^78^. Prior to fusion, two apposed lipid bilayers must approach. When the distance between the approaching bilayers is within 1 to 3 nm, a high hydration repulsion arises and dominates the interactions between them^79^, thus preventing the membranes to make contact and to proceed to fusion. Therefore, molecules that have the ability to overcome this hydration barrier could succeed in promoting membrane fusion. Bilayer dehydration can be accomplished by different mechanisms depending on the molecular nature of the fusogenic molecule^80^,^81^,^82^. Particularly, HIV-1 gp41 and influenza HA fusion peptides promote membrane dehydration by increasing the ordering of the lipid bilayers^46^,^47^. That conclusion was inferred, though, from the aforementioned S_0_/dehydration correlation and not by actually measuring the water content inside the bilayer. Our ESEEM experiments, on the other hand, have undoubtedly shown that partial membrane dehydration actually takes place upon binding of the SARS-CoV fusion peptides to POPC/POPG membranes. This finding also provided a direct evidence of the correlation between head group ordering and membrane dehydration. However, in contrast to our ESEEM data, Guillén *et al* have suggested that SARS_FP_ increases the water penetration depth into zwitterionic and anionic large unilamellar vesicles^22^. This conclusion was based on the analysis of the fluorescence decay of diphenylhexatriene (DPH) embedded in membranes, whose multi-component lifetimes were shortened in the presence of the peptide. Although the increase of water penetration depth is a possible interpretation for their results^83^, since the quantum yield of DPH decreases in water, probe location and orientation might also influence the fluorescence lifetime of DPH in lipid bilayers. Conflicting results in the literature have indicated there is no consensus yet regarding the exact location of DPH in pure model membranes^84^,^85^,^86^,^87^. Particularly, Konopásek and coworkers^86^,^87^ have shown that the short-lived component of DPH embedded in lipid bilayers originates from a probe population located at the membrane–water interface. Therefore, the subnanosecond short-lived component of DPH in the very ordered gel phase of dimyristoyl phosphatidylglycerol (DMPG) found by Guillen *et al*.^22^ in the absence of SARS_FP_ could thus potentially be due to a shallower interfacial location of DPH in the DMPG bilayer. Thus, the decrease of the DPH lifetime in the presence of SARS_FP_ found by Guillen and coworkers could potentially stem from the contact of the fluorophore with water molecules at the membrane–water interface due to a reorientational distribution of DPH in the bilayer upon the condensing effect induced by the peptide.

Lastly, it is important to emphasize that the alterations of the bilayer structure observed for SARS_FP_ and SARS_IFP_ take place at high peptide concentrations and thus are most likely due to a cooperative behavior of membrane-bound self-associated β-sheet peptides. In fact, both peptides present membrane fusion activity only at high peptide-to-lipid molar ratio and regular extended β-sheet aggregates are the most populated membrane-bound peptide conformation^15^,^22^,^23^. Peptide oligomerization in membranes as extended β-like aggregates seems to play an important role in peptide-induced membrane fusion^21^. Peptide self-association in small areas of the membrane surface might actually be important in the very first stages of the membrane fusion process^88^. Indeed, since a high amount of work is required to merge large surface areas of two lipid bilayers, lipid merging most likely proceeds through local points of contact^40^,^79^,^88^. Thus, bending moments promoting negative curvature and membrane dehydration may not only help to decrease the hydration barrier between the proximal leaflets of the approaching bilayers but also minimize the work necessary for merging the monolayers. The latter is a consequence of the possible formation of point-like membrane protrusions, also referred to as local ‘nipples’, a critical step that theoretically precedes the formation of the intermediate hemifusion stalk^40^,^79^,^88^.

### Peptides change membrane curvature of non-bilayer lipids in opposing ways

Accumulating evidence suggests that membrane fusion involves the formation of strongly curved lipid bilayers in the pre-fusion, intermediate, and post-fusion states^16^,^18^. It is therefore tempting to investigate the potential ability of fusion peptides to generate membrane curvature, since that information can help to elucidate the role played by those molecules in the viral membrane fusion process. DSC experiments with non-bilayer-forming lipids such as phosphatidylethanolamines (PE) have been successfully used to indirectly probe changes in the intrinsic spontaneous curvature properties of PE vesicles by a wide variety of peptides^75^,^89^,^90^. Shift of the L_α_-to-H_II_ transition temperature is an excellent indicator of lipid phase changes and curvature alterations^75^. We found that both SARS_FP_ and SARS_IFP_ peptides have the ability to bend DiPoPE lipid bilayers. While SARS_FP_ induces positive curvature, SARS_IFP_ causes opposing stresses on the membrane depending on the ionic strength: it promotes positive (negative) curvature strain at low (high) ionic strength. The capacity of the SARS fusion peptides to generate different curvature stresses on PE vesicles might actually be important in the context of the whole SARS-CoV S2-mediated membrane fusion.

The small stressed protrusions formed in the pre-fusion state are characterized by lipid domains possessing positive curvature flanked by negatively-curved lipid patches that stabilize the local ‘nipple’ and allow for the establishment of close intermembrane contact^16^,^79^,^88^. Therefore, both peptides can hypothetically act in this early stage by stabilizing the two curved domains depending on the ionic strength of the environment and on the exposure to PE or to negatively charged lipids^91^.

Membrane fusion proceeds with the formation of the so-called hemifusion intermediate or lipidic stalk, which is characterized by lipid mixing between the outer leaflets of the two apposed bilayers with the distal monolayers remaining unfused. The resultant mixed outer leaflet presents a high degree of negative curvature^16^,^88^. Stabilization of this intrinsic negative curvature has been traditionally interpreted as the common role played by various fusion peptides in the viral membrane fusion process^38^,^65^. The hemifusion intermediate would then subsequently progress to the formation of the fusion pore state^16^,^88^, which is characterized by lipid mixing of both the outer and inner leaflets of the merged bilayers, thus establishing the opening of a pore that allows content mixing between the two apposed membranes. In this pore state, both monolayers present strong and opposite curvature strains^16^,^88^. Based on our findings, the outer, negatively-curved monolayer of both the lipidic stalk and the fusion pore states may be stabilized by the SARS fusion peptides depending on the lipid composition: SARS_FP_ and SARS_IFP_ could act in membranes containing anionic lipids, and SARS_IFP_ could play a major role in PE-rich membranes at high ionic strength. On the other hand, the intrinsic positive curvature of the inner leaflet of the pore state can be stabilized by SARS_FP_ in the presence of PE lipids. That is, the more fusogenic SARS_FP_ peptide can also support membrane fusion by stabilization of porous structures. Experimental and computational studies have also indicated stabilization of the pore state by fusion peptides from influenza HA^37^,^92^ and parainfluenza virus (PIV5) F protein^93^. Thus, our findings imply that both SARS fusion peptides can act at different stages of the fusion process to facilitate membrane fusion.

### Putative SARS-CoV membrane fusion model

Taken together, our results reveal a functional plasticity of SARS_FP_ and SARS_IFP_ in helping to promote membrane fusion. Both peptides can act in the early and late stages of the membrane fusion reaction by changing three properties of lipid bilayers, namely spontaneous curvature, hydration, and lipid packing density. Incorporation of our findings into the currently proposed membrane fusion model induced by class I viral fusion proteins yields the following. Upon receptor binding, S2 domain of SARS-CoV Spike glycoprotein undergoes a large conformational change that releases and exposes SARS_FP_ and SARS_IFP_ to interact with target membranes^18^. Both peptides insert into but not significantly disturb (our data and data in references 15, 22 and 23) the highly-ordered, zwitterionic outer leaflet of the plasma membrane bilayer^91^. This binding process bridges viral and cell membranes and thus facilitates trimerization of other S2 subunits. Trimers are, in general, the fusion-active oligomeric state of class I fusion proteins^16^. As a result, a trimeric extended prehairpin conformation is formed. At this point, it is important to emphasize that the actual conformational state of the SARS-CoV fusion peptides remains elusive. SARS_FP_ and SARS_IFP_ adopt, respectively, a V-shaped and a linear helical conformation in dodecylphosphatidylcholine micelles^94^, but have a high tendency to aggregate and to form intramolecular β-sheets and extended β-strands stabilized by intermolecular interactions in models of lipid bilayers as well as to adopt, in small fractions, α-helical and unordered structures^22^,^23^. In the context of the intact protein, however, the structure and oligomerization state of those peptide segments still need to be addressed, although it has been proposed that membrane-bound self-associated peptides may provide the major driving force for trimerization of the whole protein^18^,^75^. Peptide binding, conformational change and possibly aggregation into the membrane may trigger S2 refolding into a trimeric hairpin conformation. As a result, a six-helix bundle would form, bringing not only viral and target membranes into close proximity, but also the internal fusion peptide (SARS_IFP_) and the pretransmembrane (SARS_PTM_) domain of the S2 subunit^95^. Due to the high hydration repulsion of the closely apposed lipid bilayers and the requirement for bending membranes to minimize areas of strong interbilayer repulsion^16^,^96^, displacement of water molecules from the membrane surface and changes in membrane curvature seem to be the prerequisites for allowing close intermembrane contact and subsequent formation of the high-energy hemifusion intermediate state. Interaction of SARS_FP_ and SARS_IFP_ with PE or with negatively charged lipids contained either in the plasma (via nonendocytic pathway) or in the endosome (via endocytic pathway) membranes may be important for the formation of point-like protrusions or for stabilization of the hemifusion stalk^91^. Since anionic phospholipids are mostly located in the inner leaflet of the membrane, it would be possible that the action of lipid flippases and scramblases could be endorsed by the peptide perturbation on the outer leaflet of the plasma membrane^97^. The major effects of the peptides at the pre-fusion state could be the following: induction of positive curvature on PE-rich membranes, as indicated by our DSC data; and membrane dehydration and induction of bending moment on the outer leaflet of bilayers comprised of anionic lipids, as suggested by our ESR data. The latter effects may also be responsible for triggering stalk formation, which is further stabilized by exposure of PE on the outer leaflet to SAR_IFP_. Hemifusion could be further facilitated by membrane interaction of a loop peptide segment located in between HR1 and HR2 domains^74^ and by a possible heteroligomerization of SARS_IFP_ with SARS_PTM_^95^. Juxtaposition of SARS_IFP_ and SARS_PTM_ leads to a synergistic and cooperative action of both peptides that causes membrane destabilization and further peptide insertion^73^. Exposure of SARS_FP_ to the inner leaflet of the merged viral and cell membranes could have a great impact in the post-fusion state. Indeed, SARS_FP_ could act by promoting positive curvature and stabilizing the high positively-curved inner leaflet that characterizes the porous state, thus facilitating pore formation (our DSC data). However, the molecular details of the above processes still need to be investigated. Overall, the two putative fusion peptides from SARS-CoV S protein may help to regulate membrane fusion by acting in the early and late stages of the membrane fusion process.

## Conclusions

Our main findings were: (1) SARS fusion peptides increase the ordering of the headgroup and acyl chain regions of MLVs containing negatively-charged, but not zwitterionic phospholipids; (2) Membrane fusion promoters induce similar effects on the head group ordering than do the fusion peptides, whereas membrane fusion inhibitors cause opposing effects; (3) Changes in the order parameters of the lipids are generally greater for the more fusogenic SARS_FP_ peptide than for SARS_IFP_; (4) Both peptides promote dehydration of PG-containing membranes and this effect is well correlated with the increased head group ordering; and (5) DSC data support a hypothesis that SARS_FP_ induces positive curvature on DiPoPE vesicles, whereas SARS_IFP_ promotes opposing stresses on the intrinsic negative curvature of DiPoPE depending on the ionic strength.

Peptide-induced chain-packing energy and membrane surface ordering of the outer leaflet of negatively charged lipid bilayers promote partial membrane dehydration and could generate bending moment, as suggested by our ESR studies. Both effects may induce negative curvature and decrease the hydration repulsion of apposed bilayers. Possible peptide involvement on the formation of the pre-fusion point-like protrusions and intermediate hemifusion stalk as well as on the stabilization of the fusion pore state suggest that the SARS fusion peptides might play important roles in the whole membrane fusion process. Taken together, our findings suggest that the SARS fusion peptides have the ability to change the physicochemical properties of model membranes depending on the lipid composition and on the ionic strength. Therefore, they can act in the early and late stages of the membrane fusion process, conferring them a functional plasticity that might be important to help overcome the high kinetic barrier involved in the SARS-CoV-induced membrane fusion.

## Methods

### Materials

N-terminally acetylated and C-terminally amidated SARS_FP_ (^770^MWKTPTLKYFGGFNFSQIL^788^) and SARS_IFP_ (^873^GAALQIPFAMQMAYRF^888^) peptides were either purchased from GenScript (Piscataway Township, NJ) or manually synthesized according to the standard Fmoc solid-phase peptide synthesis method on a Rink-Amide resin^98^. The details of peptide synthesis are described in Vicente *et al*.^99^. Purification was performed as described in Supplementary section SI1.

The phospholipids 1-palmitoyl-2-hydroxy-sn-glycero-3-phosphocholine (LPC), 1,2-dipalmitoyl-sn-glycero-phosphatidylcholine (DPPC), 1,2-dipalmitoyl-sn-glycero-3-phospho-(1’-rac-glycerol) (DPPG), 1,2-dipalmitoyl-sn-glycero-3-phospho-L-serine (DPPS), 1,2-dipalmitoleoyl-sn-glycero-3-phosphoethanolamine (DiPoPE), 1-palmitoyl-2-oleoyl-sn-glycero-3-phosphocholine (POPC), 1-palmitoyl-2-oleoyl-sn-glycero-3-phospho-(1’-rac-glycerol) (POPG), 1-palmitoyl-2-oleoyl-sn-glycero-3-phospho-L-serine (POPS), 1-palmitoyl-2-oleoyl-sn-glycero-3-phosphate (POPA), and the spin labels 1-palmitoyl-2-stearoyl(n-doxyl)-sn-glycero-3-phosphocholine (n-PCSL, where n = 5 and 16), 1,2-dioleoyl-sn-glycero-3-phospho(tempo)choline (DOPTC), and 1,2-dipalmitoyl-sn-glycero-3-phospho(tempo)choline (DPPTC) were purchased from Avanti Polar Lipids, Inc. (Alabaster, AL). Cholesterol (Chol) and linoleic acid (LA) were obtained from Sigma-Aldrich (St. Louis, MO). All reagents were used without further purification.

### Sample preparation

Phospholipids (1.6 mg for DSC and 0.5 mg for ESR) and spin labels (0.5 mol% for CW ESR and 1 mol% for pulsed ESR) either in chloroform or chloroform/methanol 1:1 (v/v) stock solutions were mixed in a glass tube. After dried under a N_2_ flow, the lipid film was ultracentrifuged under vacuum overnight to remove traces of solvent. For CW ESR experiments, the sample was hydrated in 20 mM potassium phosphate buffer, pH 7.4, sonicated in a bath type sonicator for a few seconds and maintained at a temperature above the main phase transition of the lipid for at least two hours for complete hydration. Samples were then subjected to at least six freeze-thaw cycles. A measured volume of SARS_FP_ or SARS_IFP_ stock solutions in dimethyl sulfoxide (DMSO) was added to the preformed multilamellar lipid dispersions. For DSC experiments, peptides dissolved in either acetonitrile/water 1:1 (v/v) or in DMSO solutions were diluted into buffer and added to the lipid film for hydration. Samples were vortexed for few seconds, maintained at a temperature above the phase transition for each lipid during at least 30 min, and subjected to six freeze-thaw cycles. The amount of phospholipid (final lipid concentration of 10 mg/ml for ESR and 2 mg/ml for DSC) and peptides used provided a 20:1 lipid/peptide molar ratio for most of the experiments. It is worth mentioning that the same amount of DMSO or acetonitrile/water 1:1 added in the peptide-containing samples was also used in the peptide-free samples as controls for the ESR and DSC experiments. The control samples were prepared using the same protocol as those of the peptide-containing samples. For DiPoPE/peptide samples, peptides and lipids dissolved in chloroform/methanol 1:1 (v/v) stock solutions were mixed in a glass tube, dried to a lipid film under N_2_ gas and lyophilized overnight. Samples were hydrated in 20 mM sodium phosphate buffer, pH 7.4, with or without 150 mM sodium chloride, and freeze-thaw cycled six times below the liquid crystalline-to-inverted hexagonal (Lα-H_II_) phase transition temperature (T_H_) of the lipid. DiPoPE concentration was 10 mg/ml and peptide concentration varied from 0.2 to 0.5 mol% (500:1 and 200:1 lipid/peptide molar ratio, respectively). For ESEEM experiments, POPC/POPG 7:3 mol/mol and peptides (20:1 lipid/peptide molar ratio) were prepared as above, but hydrated in 20 mM sodium phosphate, 150 mM NaCl D_2_O buffer, pD = 7.4 (actual pH measurement). Peptide concentration was confirmed spectrophotometrically by using the theoretical molar extinction coefficients of 6,990 M^−1^cm^−1^ for SARS_FP_ and 1,490 M^−1^cm^−1^ for SARS_IFP_.

### DSC Experiments

The effects of the peptides on the thermotropic behavior of the lipid phase transitions were recorded in a VP-DSC MicroCal MicroCalorimeter (Microcal, Northampton, MA, USA) using a heating rate of 33.2 °C/h for DiPoPE and of 23.4 °C/h for the other lipids. Samples were firstly degassed and then equilibrated for 15 minutes at the starting temperature prior to measurements. Analyses of thermograms were performed using Microcal Origin software.

### ESR Experiments

CW-ESR experiments were carried out on a Varian E-109 spectrometer operating at 9.5 GHz. Temperature was controlled by a homemade temperature control unit coupled to the spectrometer, whose accuracy is about 0.2 °C. Samples were transferred to glass capillaries (1.5 mm I.D.), which were set into a quartz tube containing a mineral oil bath to help stabilize the sample temperature. The following acquisition parameters were used: center field, 3,362 G; scan width, 80 to 160 G; modulation amplitude, 0.5 or 1.0 G; modulation frequency, 100 kHz; microwave power, 5 or 10 mW; time constant, 128 ms, and acquisition time, 240 s.

Nonlinear least-squares simulations (NLLS) of the CW-ESR spectra were performed using the Multicomponent LabView (National Instruments) software developed by Dr. Christian Altenbach (University of California, Los Angeles, California)^24^,^100^. The rotational diffusion rates (R_⊥_, R_∥_) and order parameters (S_0_, S_2_) were obtained as described in ref. 33 with further details in the section SI2 of supplementary information. Seed values for the magnetic parameters of both 5-PCSL and 16-PCSL were obtained from Earle *et al*.^44^ and those of DPPTC were taken from Ge and Freed^101^. The strategy of the NLLS simulation was performed as described elsewhere^33^.

Pulsed ESR experiments were performed on a Bruker Elexsys 580 X-band pulsed ESR spectrometer equipped with the Bruker Flexline ER 4118X-MS3 split-ring resonator and the ITC503 Oxford cryogenic system for temperature control. Samples were immersed into liquid nitrogen prior to the measurements at 50 K. Three pulse electron spin echo envelope modulation (ESEEM) experiments were carried out with the π/2 – τ – π/2 – T – π/2 – τ – echo pulse sequence^54^ and using a four-step phase cycling to suppress unwanted echoes^102^. The microwave power was adjusted to give 16 ns π/2 pulses and an interpulse delay *τ* of 236 ns, kept constant in all experiments, was chosen to maximize deuterium modulations at the magnetic field where the echo intensity is maximum. Starting at time delay T = 200 ns, 700 points were recorded with ΔT = 12 ns steps to obtain the three-pulse stimulated echo decays. The integration gate length was 48 ns and the shot repetition time was 1,500 μs. The number of accumulations varied from 20 to 50 depending on the signal-to-noise ratio and on the modulation depth. Data analysis was performed as described in Bartucci *et al*.^62^. Briefly, the contribution of the spin relaxation to the ESEEM signal was eliminated by dividing the time-dependent echo amplitudes, V(τ, T), by a bi-exponential decay, 〈V(τ, T)〉, followed by subtraction of unity, as V_norm_(τ, T) = V(τ, T)/〈V(τ, T)〉 − 1. The remained oscillations about zero were apodized with a Hamming window and zero-filled to increase the total number of points to about 4 K. Numerical Fourier transformation was performed and the resultant magnitude spectrum was multiplied by the dwell time ΔT = 12 ns to provide a spectral density in ns units.

## Additional Information

**How to cite this article**: Basso, L. G. M. *et al*. SARS-CoV fusion peptides induce membrane surface ordering and curvature. *Sci. Rep.* **6**, 37131; doi: 10.1038/srep37131 (2016).

**Publisher's note:** Springer Nature remains neutral with regard to jurisdictional claims in published maps and institutional affiliations.

## Supplementary Material

Supplementary Information

## Figures and Tables

**Table 1:** Thermodynamics parameters obtained from DSC experiments of lipid phase transitions in the absence and presence of 5 mol% of fusion peptides at low ionic strength.

Sample	ΔH (kcal/mol)	T_m,1_ (°C)	ΔT_1/2,1_ (°C)	T_m2_ (°C)	ΔT_1/2,2_ (°C)	ΔS (cal/mol K)	T_P_ (°C)
*DPPC*							
Blank	9.41	40.6	0.15	—	—	29.9	32.9
SARS_FP_	9.64	41.0	0.14	—	—	30.7	34.4
SARS_IFP_	9.46	40.8	0.13	—	–	30.1	33.9
*DPPG*							
blank	9.40	39.8	0.36	—	—	30.0	30.9
SARS_FP_	4.57	39.6	0.38	—	—	14.6	31.0
SARS_IFP_	5.73	39.9	0.63	—	—	18.3	32.2
*DPPG (salt)*							
blank	9.31	40.0	0.56	39.2	—	29.7	33.0
SARS_FP_	6.08	40.0	0.50	—	—	19.4	33.0
SARS_IFP_	4.41	40.1	0.61	–	—	14.1	33.9
*DPPS*							
blank	6.36	51.1	0.48	52.2	0.58	—	—
SARS_FP_	5.42	51.1	0.24	52.0	0.42	—	—
SARS_IFP_	4.86	51.1	—	52.1	0.78	—	—
*POPA*							
blank	6.37	23.2	1.42	—	—	21.5	—
SARS_FP_	5.88	23.2	—	21.1	—	—	—
SARS_IFP_	5.43	23.7	—	21.5	–	—	—
*POPE*							
blank	5.38	24.6	2.94	—	—	18.1	—
SARS_FP_	4.04	24.4	4.08	—	—	13.6	—
SARS_IFP_	4.52	23.6	2.97	—	—	15.2	—
*POPS*							
blank	5.44	10.0	1.17	—	—	19.2	—
SARS_FP_	2.79	10.7	1.00	11.9	—	—	—
SARS_IFP_	3.08	10.6	2.03	12.4	—	—	—

The parameters from DPPG samples at 150 mM NaCl (*salt*) are also shown. T_P_ and T_m_ stand for the pretranstion and main phase transition temperatures, respectively, ΔT_1/2_ represents the linewidth at half height of the main transitions, ΔH represents the calorimetric enthalpy changes over all transitions observed and ΔS corresponds to the entropy change of the main phase transition.

Estimated uncertainties: ΔH (~3%), T_m_ (0.1 °C), T_P_ (0.4 °C), ΔT_1/2_ (0.02 °C).

**Table 2:** Thermodynamics parameters of the Lα-to-H_II_ phase transition of peptide-free and peptide-containing DiPoPE liposomes at low (0 mM NaCl) and high (150 mM NaCl) ionic strength.

Sample	ΔH_H_ (cal/mol)	T_H_ (°C)	ΔT_1/2_ (°C)
*DiPoPE (0* *mM NaCl*)	136	44.3	2.0
+0.2 mol% SARS_FP_	91	47.9	2.4
+0.5 mol% SARS_FP_	74	50.2	3.7
+0.2 mol% SARS_IFP_	92	45.4	1.7
+0.5 mol% SARS_IFP_	40	44.6	2.2
*DiPoPE (150* *mM NaCl*)	107	47.5	2.3
+0.2 mol% SARS_FP_	101	48.7	3.1
+0.5 mol% SARS_FP_	44	50.1	3.4
+0.2 mol% SARS_IFP_	83	43.7	2.5

Estimated uncertainties: ΔH_H_ (8–15%), T_H_ (0.2 °C), ΔT_1/2_ (0.3 °C).

The high inaccuracy on the values of ΔH_H_ was mainly due to extensive lipid aggregation of peptide-free and peptide-containing DiPoPE vesicles from different sample preparations. However, not only the T_H_ was reproducible from different samples, but also the general trend of decreasing the ΔH_H_ of the transition in the peptide-containing DiPoPE samples was observed in all experiments.
