# Supplementary material for: SARS-CoV fusion peptides induce membrane surface ordering and curvature
Source: Sci Rep. 2016 Nov 28;6:37131. doi: 10.1038/srep37131 (PMC5125003; doi:10.1038/srep37131)
Supplement: Supplementary Information [file srep37131-s1.doc]

**Supplementary Information**

**SARS-CoV fusion peptides induce membrane surface ordering and curvature**

Luis G. M. Basso^1,2^, Eduardo F. Vicente^3^, Edson Crusca^4^, Eduardo M. Cilli^4^, and Antonio J. Costa-Filho^2,*^

^1^Grupo de Biofísica Molecular Sérgio Mascarenhas, Instituto de Física de São Carlos, Universidade de São Paulo, Avenida Trabalhador São-carlense, 400, Centro, São Carlos, SP, Brazil.

^2^Laboratório de Biofísica Molecular, Departamento de Física, Faculdade de Filosofia, Ciências e Letras de Ribeirão Preto, Universidade de São Paulo. Av. Bandeirantes, 3900, 14040-901, Ribeirão Preto, SP, Brazil.

^3^Faculdade de Ciências e Engenharia, UNESP – Univ Estadual Paulista, Campus de Tupã. Rua Domingos da Costa Lopes, 780, 17602-496, Tupã, SP, Brazil.

^4^Departamento de Bioquímica e Tecnologia Química, Instituto de Química, UNESP – Univ Estadual Paulista. Rua Prof. Franscisco Degni, 55, 14800-900, Araraquara, SP, Brazil.

*Corresponding Author. Email address: [ajcosta@ffclrp.usp.br](mailto:ajcosta@ffclrp.usp.br). Phone: (+55)163315-3665.

**Supplementary Methods**

## SI1. Purification of the synthetic peptides

Purification of the crude peptides was performed by a semi-preparative HPLC on a Shimadzu system (Japan) using a reverse phase C_18_ column with a linear gradient 20-50% of solvent B (0.036% v/v TFA/acetonitrile) for 90 min. The flow rate was 5 mL/min and UV detection at 220 nm. Peptide purity was checked by analytical HPLC (Varian, USA), using solvents A (0.045% v/v TFA/H_2_O) and B with a linear gradient of 5-95% v/v of solvent B for 30 min, at a flow rate of 1.0 mL/min and UV detection at 220 nm. The identity and characterization of the peptides were confirmed by electrospray mass spectrometry on a ZMD model apparatus (Micromass, UK). The synthetic peptides were used with purity higher than 95%.

**SI2. Nonlinear least-squares (NLLS) ESR simulations of the CW ESR spectra**

The rotational dynamics and ordering of the spin-labeled lipids are characterized by two sets of parameters: (R_⊥_, R_∥_), which are the principal components of the rotational diffusion tensor; and (S_0_, S_2_), which are the ordering tensor parameters. The first set describes the dynamics of the spin-labeled molecule through rates of rotational diffusion of the nitroxide moiety around axes parallel (R_∥_) and perpendicular (R_⊥_) to the preferential orienting axis of the acyl chain or of the lipid headgroup. The second set, S_0_ and S_2_, describes the microscopic or local orientational ordering of the spin label. S_0_ represents the average angular amplitude of the lipid segment to which the nitroxide is attached and S_2_ is a measure of the molecular nonaxiality of the motion of the spin label. Thus, for spin-labeled lipids, S_0_ indicates how well the lipid segment is aligned along the local director of the membrane, i.e., the normal vector to the lipid bilayer, whereas S_2_ represents the deviation from cylindrical symmetry of the molecular alignment to the local director. These two parameters are defined as follows:

| $S_{0}=\left\langle D_{0,0}^{2}\right\rangle=\left\langle\frac{3\cos^{2}\theta-1}{2}\right\rangle=\frac{\int d\Omega D_{0,0}^{2}\exp\left(-\frac{U(\Omega)}{k_{B}T}\right)}{\int d\Omega\exp\left(-\frac{U(\Omega)}{k_{B}T}\right)}$ |
| --- |
| $S_{2}=\left\langle D_{0,2}^{2}+D_{0,-2}^{2}\right\rangle=\left\langle\sqrt{\frac{3}{2}}\sin^{2}\theta\cos(2\varphi)\right\rangle$ |

where k_B_ is the Boltzmann’s constant and T is the temperature. The restoring potential U(Ω) governs the tendency of the spin label to align relative to the local director and thus restricts the amplitude of its rotational motion. It is conveniently written in a series of generalized spherical harmonics $D_{0,K}^{L}$^1^:

| $-\frac{U(\theta,\varphi)}{k_{B}T}=\sum_{\begin{matrix} 0<L,K\leq2 \\ even\mathop{}\limits L,K \end{matrix}} c_{LK}\left[D_{0,K}^{L}(\theta,\varphi)+D_{0,-K}^{L}(\theta,\varphi)\right]=c_{20}\left(\frac{3\cos^{2}\theta-1}{2}\right)+c_{22}\sqrt{\frac{3}{2}}sen^{2}\theta\cos2\varphi$ |
| --- |

where $\Omega\equiv(\theta,\phi)$ are the polar angles of the local director in the rotational diffusion axis frame and the dimensionless coefficients c_20_ and c_22_ are the parameters to be minimized during the fitting process. Because the local directors of the membranes in the sample are not aligned with each other, the locally ordered domains are randomly oriented with respect to the direction of the external applied magnetic field (laboratory frame). Therefore, the microscopic order with macroscopic disorder (MOMD) model^2^ was used to simulate the ESR spectra of the spin-labeled lipids in multilamellar vesicles.

**SI3. On the negative membrane curvature promoted by bending moment**

As discussed by Koller and Lohner in a nice review article on interfacially active peptides^3^, several factors have been proposed to be relevant for curvature-induction by membrane-bound peptides: H-bonding, electrostatic repulsion, monolayer surface area and lateral pressure. The main assumption of our work is that the SARS fusion peptides redistribute the lateral pressures within the headgroup and the hydrocarbon regions of the outer monolayer and thus induce a bending moment in the bilayer. The reasoning for that is given below.

According to the theory of elastic properties of lipid bilayers^4-6^, the mechanical bending moment per unit length, *M*, defined as the product of the spontaneous curvature, *c_0_*, and the bending moduli/rigidity, *κ*, depends on the first moment of the pressure profile along the bilayer normal, *π(z)*:

$M=\kappa c_{0}=\int_{-t}^{t} \pi\left(z\right)zdz$,

where z = 0 is taken at the middle of the membrane and 2t represents the thickness of the bilayer. The above equation only provides the lipid chain contribution to the bending moment and to the curvature elastic constant, but by extending the integral to the aqueous phase, one can obtain the interfacial contributions, which arise from head group repulsion and surface tension. The pressure profile across the bilayer is the result of the balance between the attractive and repulsive forces acting in different regions of the monolayer^7^. Therefore, changes in lipid packing density and in water concentration at the interfacial region can profoundly change *π(z)*, since they can enhance or decrease the attractive or the repulsive forces between the lipids.

It is clear from the equation above that for a flat bilayer possessing a symmetric pressure profile, the integral over *π(z)* along the bilayer normal must vanish and thus *M = 0*. On the other hand, an asymmetric change on *π(z)*, i.e., relative differences in the lateral pressure distribution, generates a uniform bending moment (*M ≠ 0*) that ultimately would tend to bend the membranes toward one leaflet. Note that the largest contribution to the integral above comes from the headgroup region. As discussed by Ge and Freed^8^, this is mainly due to the profile of lateral stress across the bilayer, the strength of the ionic interactions in the headgroup, and the larger values of z in the interfacial region compared to those in the hydrophobic core. Thus, the peptide-induced condensation of the outer monolayer due to increased lipid packing and headgroup ordering as well as membrane dehydration would generate bending moment that could promote negative curvature. Due to the similar results we obtained with the SARS peptides compared to those from other fusion peptides^8,9^, the abovementioned bending-moment mechanism of generating curvature stress in anionic membranes due to relative changes on the pressure profile across the bilayer could represent the putative molecular mechanism played by the SARS peptides to induce membrane fusion.

**Supplementary figures**

**Figure S1.** Lipid structures of the spin labels used in the present work. Adapted from Avanti Polar lipids website (http:// http://avantilipids.com/).


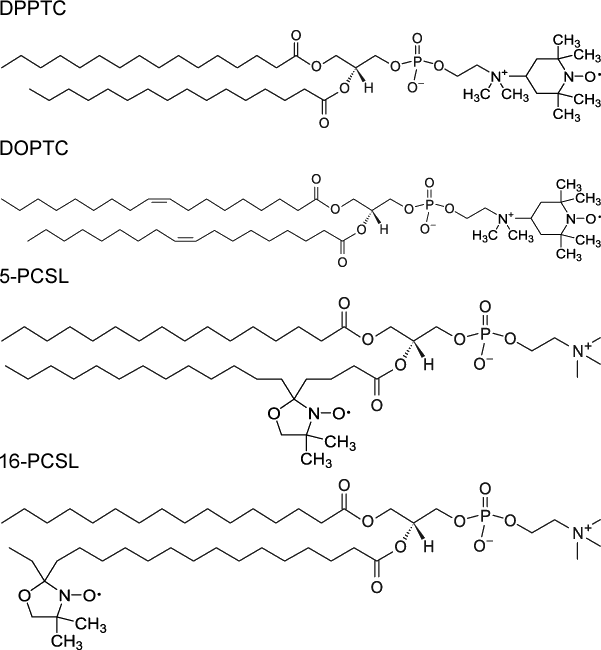


**Figure S2.** Excess heat capacity profile of the low-enthalpic, higher-temperature endothermic peak of DPPS in the absence (black) and presence of 5 mol% of SARS_FP_ (red) and SARS_IFP_ (blue) obtained from peptide stock solution in DMSO.


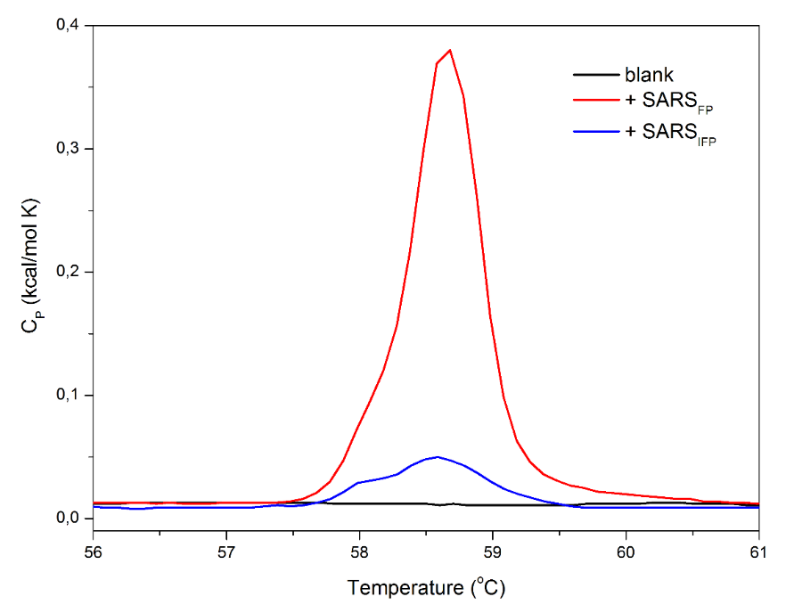


**Figure S3.** ESR spectra of DPPTC, 5-PCSL, and 16-PCSL in zwitterionic MLVs at different temperatures (25, 37, and 45^o^C) without (black) and with 5 mol% of SARS_FP_ (blue) and SARS_IFP_ (red) peptides at 1:28 amd 1:20 peptide/lipid molar ratios, respectively: (**A**) Pure DPPC and (**B**) DPPC/Chol 7:3 mol/mol.


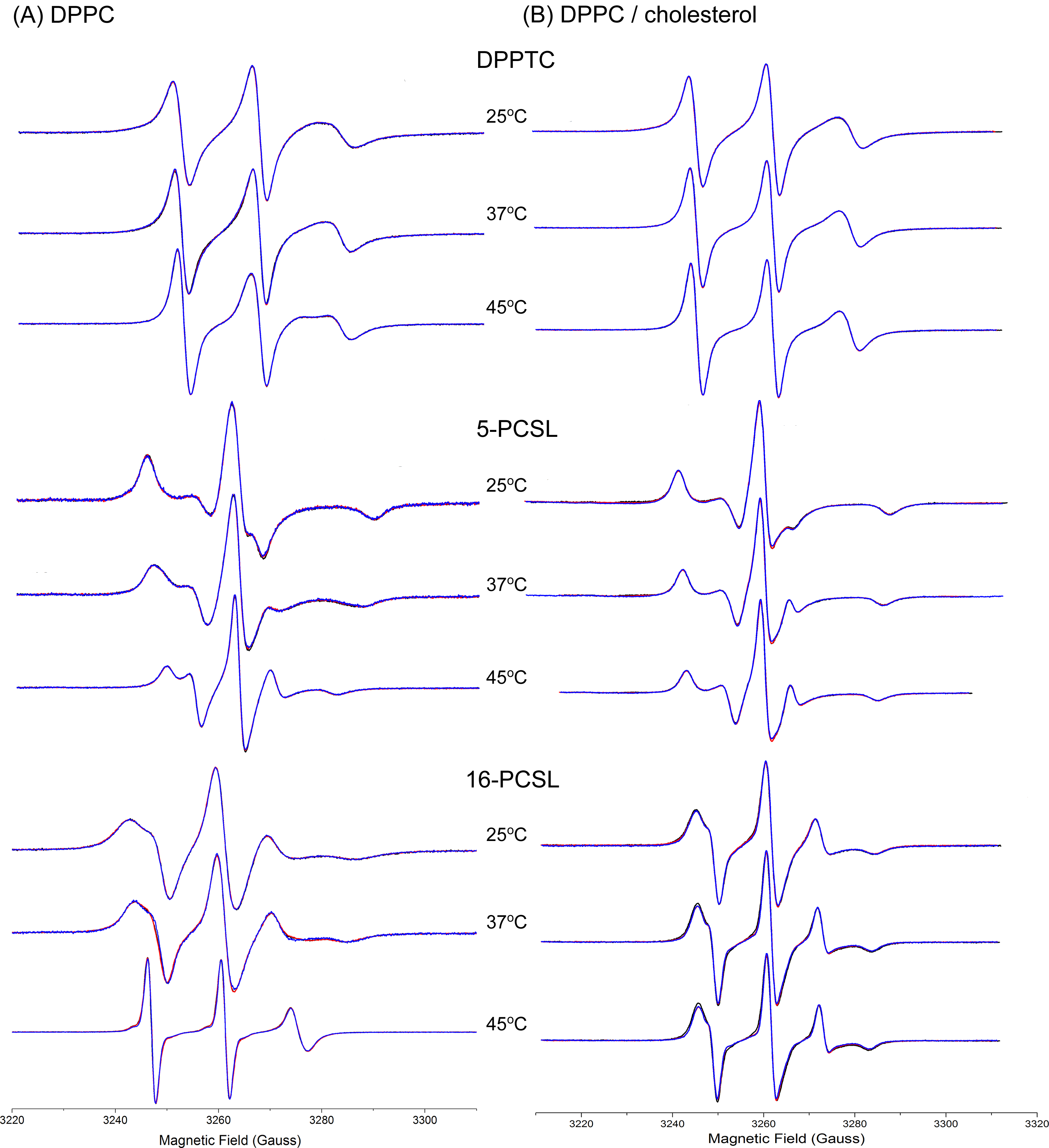


**Figure S4.** **Lineshape changes of the ESR spectra promoted by the peptides**. (**A**) Selected ESR spectra of DPPTC, 5-PCSL, and 16-PCSL in DPPG, DPPS, and POPA MLVs at different temperatures without (black) and with 5 mol% of SARS_IFP_ (red) and SARS_FP_ (blue). Spectra were normalized by the height of the center-field line. (**B**) Experimental (left) and best NLLS fits (right) of DPPTC spectra in DPPG at 37^o^C along with the fit residuals (gray). The spectral differences between the signals obtained from the peptide-free and the peptide-containing samples (SARS_IFP_ on the left and SARS_FP_ on the right) are displayed at the bottom. The good agreement between the experimental (black) and simulated (red) spectral differences indicates the goodness of the nonlinear-least-squares fits.

**
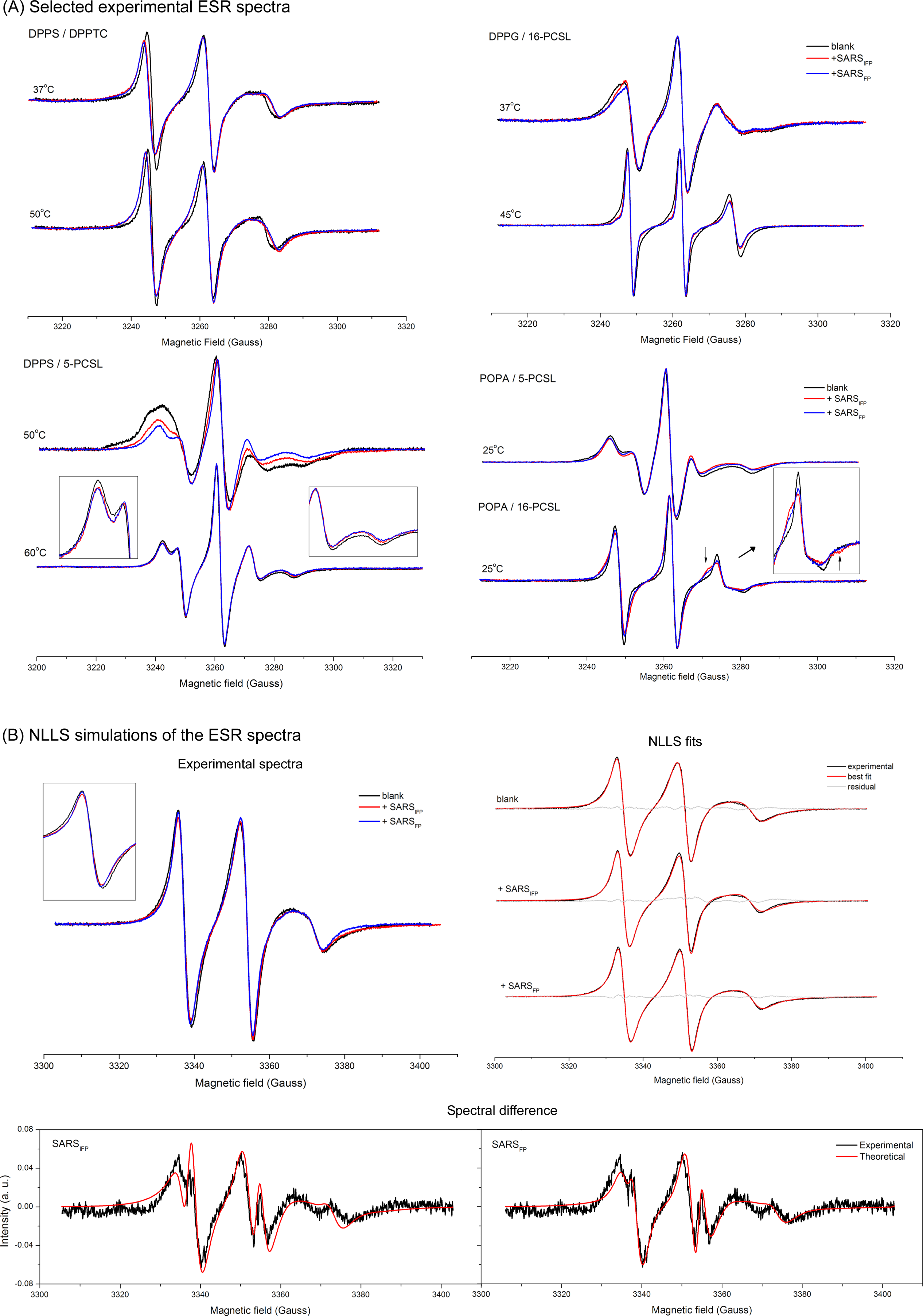
**

**Figure S5.** Experimental (solid) and best-fit (dashed) ESR spectra of DPPTC, 5-PCSL, and 16-PCSL in (**A**) DPPG, (**B**) DPPS, and (**C**) POPA MLVs at different temperatures without and with 5 mol% of SARS_FP_ and SARS_IFP_ peptides. Scan range: 140 G for 5-PCSL in DPPG and DPPS and 100 G for all the other conditions.


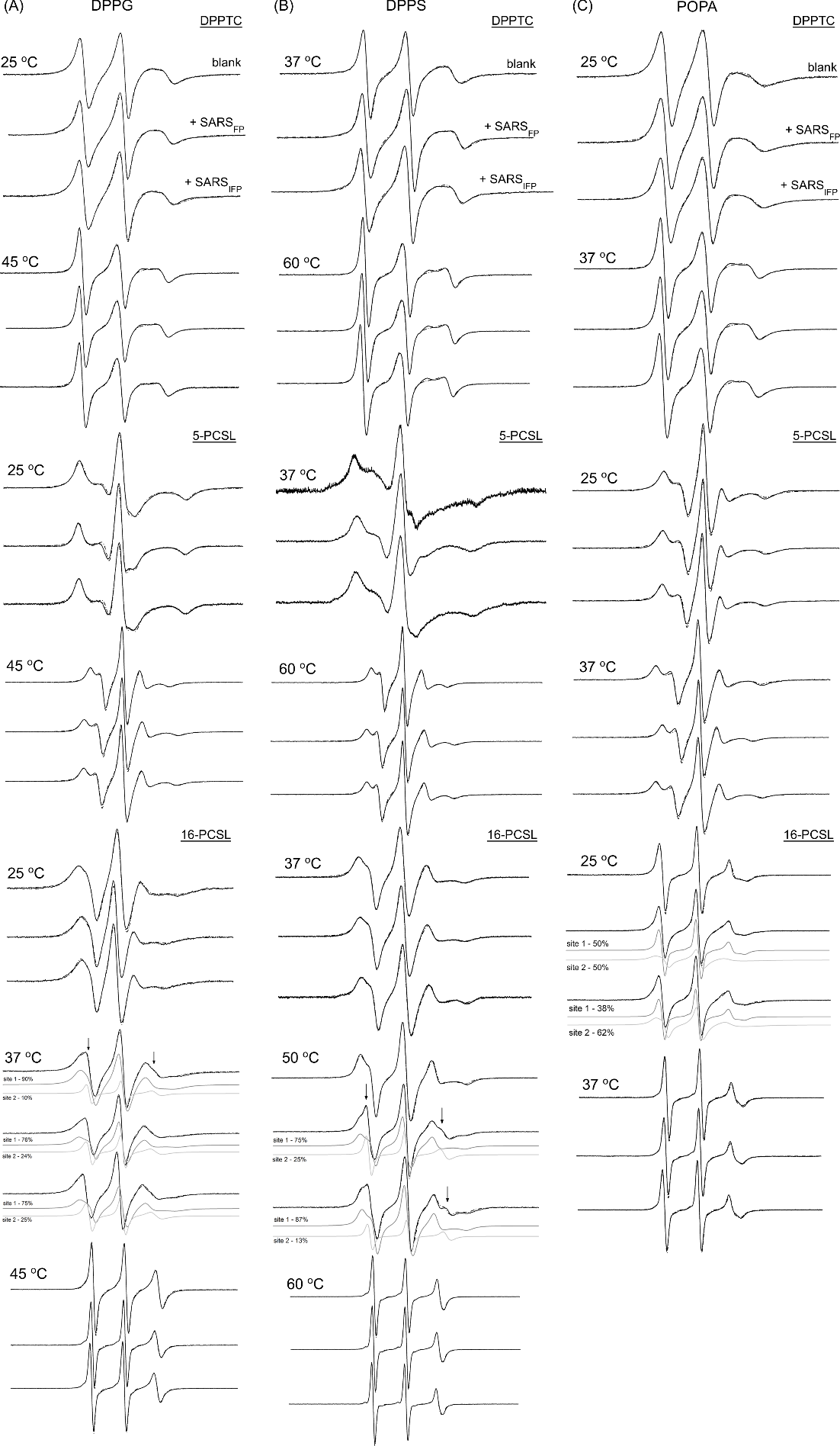


**Figure S6.** Experimental (black) and best-fit NLLS (red) ESR spectra of DPPTC in equimolar mixtures of (**A**) DPPC/DPPG and (**B**) DPPC/POPA MLVs at 37 ^o^C without and with 5 mol% of fusion peptides SARS_FP_ and SARS_IFP_ or 10 mol% of the membrane fusion promoter linoleic acid (LA) or the membrane fusion inhibitor lyso-PC (LPC). The residual signal (gray) was obtained by subtracting the theoretical spectrum from the experimental one. Scan range: 100 G.

**
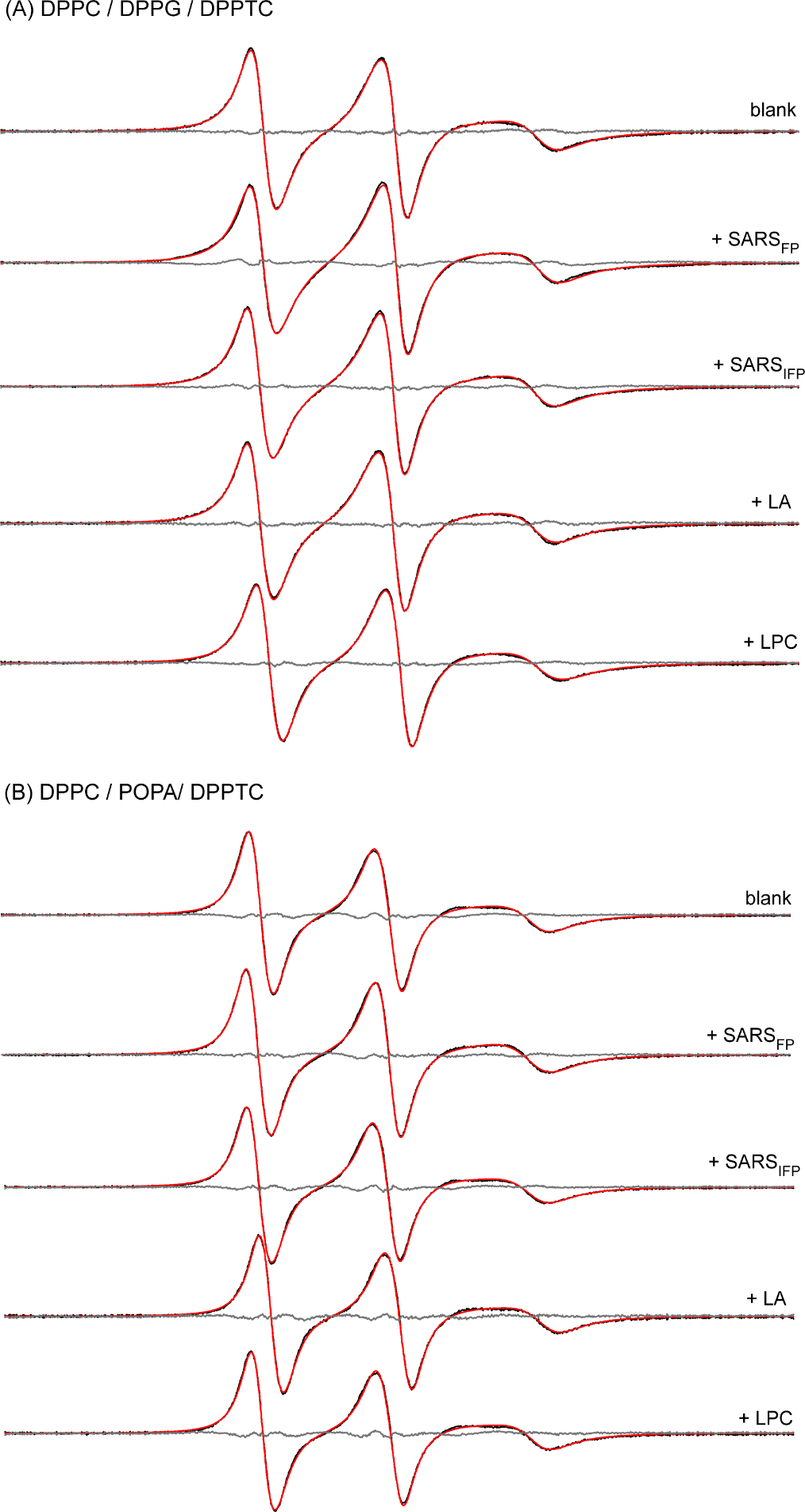
**

**Figure S7.** **Representative normalized spin echo intensities and spectral densities**. (**A**) Time-domain ESEEM spectra of DOPTC, 5-PCSL, and 16-PCSL in POPC/POPG 7/3 (mol/mol) membranes without and with 5 mol% of fusion peptides. Note the high- and low-frequency ESEEM oscillations due to proton and deuterons, respectively. (**B**) ESEEM spectra of the same spin labels as before in POPC/Chol 7/3 (mol/mol) (top) and the corresponding spectral densities (bottom). Water concentration is virtually zero from the 10^th^ carbon position along the acyl chain down to the bilayer center. For convenience, all time-domain spectra were shifted upwards by 0.2 or 0.3. (**C**) Frequency-domain ESEEM spectra of DOPTC and n-PCSL (n = 5, 16) spin labels in DPPC membranes.

**
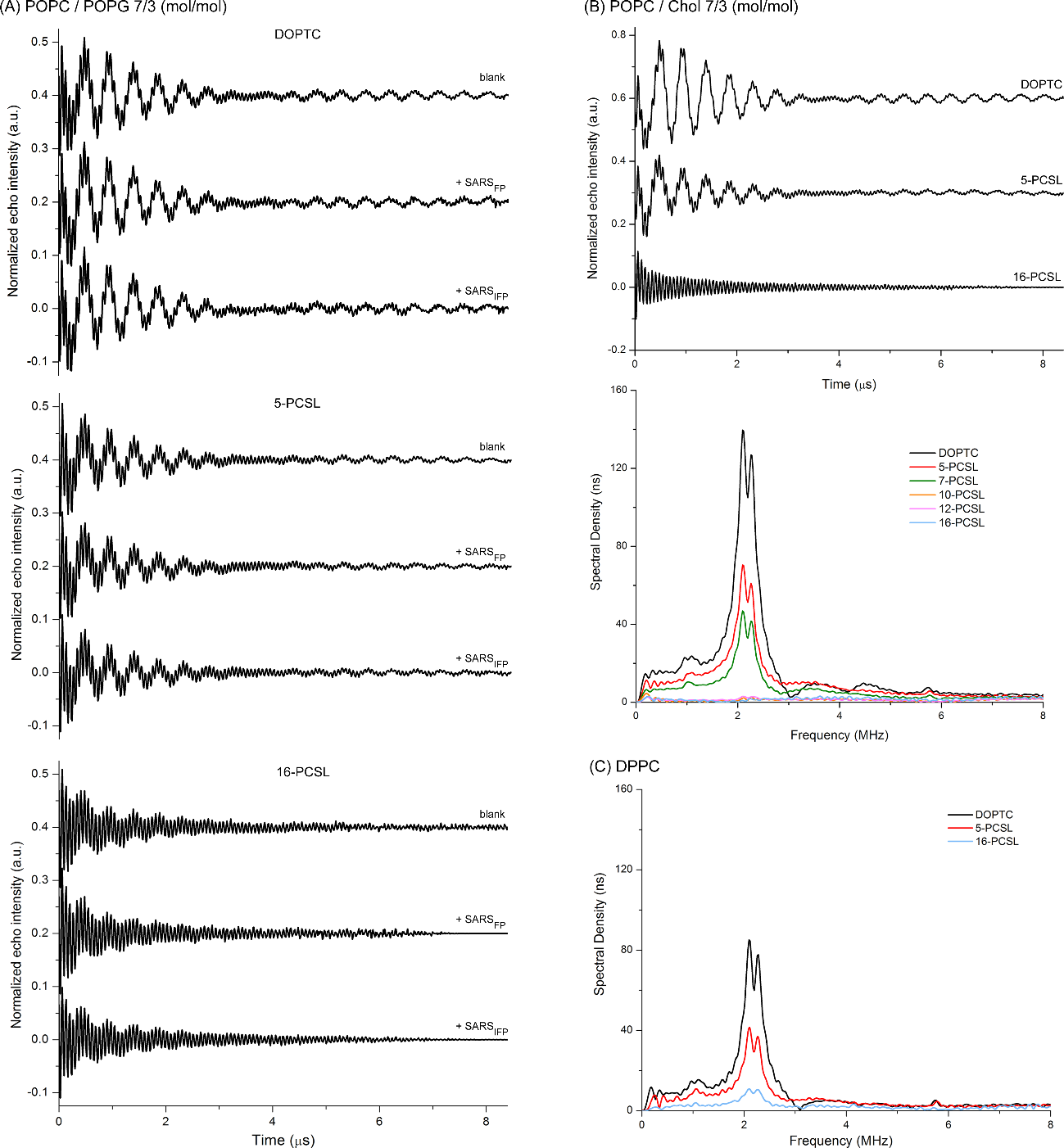
**

**Supplementary tables**

**Table S1.** Thermodynamics parameters of the low-enthalpic, higher-temperature endothermic peak of DPPS in the absence and presence of 5 mol% of SARSFP and SARSIFP. Peptide stock solution in either acetonitrile/water 1:1 or DMSO.

|  |  |  |  |
| --- | --- | --- | --- |
| Sample | ΔH_3_ (cal/mol) | T_3_ (^o^C) | ΔT_1/2_ (^o^C) |
|  |  |  |  |
|  |  |  |  |
| *DPPS (acetonitrile/water)* |  |  |  |
|  |  |  |  |
| blank | – | – | – |
| + FP | 44.6 | 57.9 | 0.46 |
| + IFP | 140.9 | 58.0 | 0.53 |
|  |  |  |  |
| *DPPS (acetonitrile/water)* |  |  |  |
|  |  |  |  |
| blank | – | – | – |
| + FP | 64.3 | 58.1 | 0.42 |
| + IFP | 174.6 | 58.3 | 0.55 |
|  |  |  |  |
| *DPPS (DMSO)* |  |  |  |
|  |  |  |  |
| blank | – | – | – |
| + FP | 274.5 | 58.6 | 0.62 |
| + IFP | 41.2 | 58.5 | 1.00 |
|  |  |  |  |

**Table S2.** G- and hyperfine-tensor components of DPPTC, 5-PCSL, and 16-PCSL in the different lipid model membranes used for NLLS simulations.

|  |  |  |  |  |  |
| --- | --- | --- | --- | --- | --- |
| System | g_xx_ | g_yy_ | g_zz_ | A_xx_ | A_yy_ |
|  |  |  |  |  |  |
|  |  |  |  |  |  |
| DPPG / DPPTC | 2.0081 | 2.0065 | 2.0021 | 6.0 | 6.0 |
| DPPG / 5-PCSL | 2.0089 | 2.0059 | 2.0025 | 5.5 | 5.5 |
| DPPG / 16-PCSL | 2.0089 | 2.0058 | 2.0025 | 4.9 | 4.9 |
|  |  |  |  |  |  |
| DPPS / DPPTC | 2.0084 | 2.0063 | 2.0020 | 6.0 | 6.0 |
| + peptides | 2.0080 | 2.0063 | 2.0023 | 6.0 | 6.0 |
| DPPS / 5-PCSL | 2.0088 | 2.0059 | 2.0021 | 4.9 | 4.9 |
| DPPS / 16-PCSL | 2.0089 | 2.0058 | 2.0025 | 4.9 | 4.9 |
|  |  |  |  |  |  |
| POPA / DPPTC | 2.0087 | 2.0061 | 2.0020 | 5.0 | 5.0 |
| POPA / 5-PCSL | 2.0088 | 2.0059 | 2.0021 | 5.0 | 5.0 |
| POPA / 16-PCSL | 2.0088 | 2.0059 | 2.0033 | 4.9 | 4.9 |
|  |  |  |  |  |  |
| DPPC / DPPG / DPPTC | 2.0084 | 2.0065 | 2.0020 | 6.0 | 6.0 |
| DPPC / POPA / DPPTC | 2.0084 | 2.0062 | 2.0020 | 6.0 | 6.0 |
|  | | | | | |
|  | | | | | |
| - g_xx_ of DPPTC in DPPG was allowed to slightly vary with temperature due to poor fitting with fixed values. This parameter changed from 2.0081 at 25^o^C to 2.0084 at 37^o^C to 2.0086 at 45^o^C and was used for simulations of both peptide-free and peptide-containing DPPG/DPPTC membranes. - g_zz_ of 16-PCSL in POPA was allowed to change to 2.0021 for the second, peptide-bound component. - g_xx_ of DPPTC slightly changed to 2.0081 in the peptide-containing DPPC/DPPG membranes. | | | | | |

**Table S3.** Best-fit rotational diffusion rates (R_┴_, R//), rotational correlation time (τ), order parameters (S_0_, S_2_), and A_zz_ component obtained from NLLS simulations of DPPTC, 5-PCSL, and 16-PCSL ESR spectra in DPPG model membranes at selected temperatures in the absence and in the presence of 5 mol% of peptides.

|  |  |  |  |  |  |  |  |
| --- | --- | --- | --- | --- | --- | --- | --- |
| System | T (^o^C) | R_┴_ (×10^7^ s^-1^) | R// (×10^8^ s^-1^) | τ (ns) | S_0_ | S_2_ | A_zz_ (G) |
|  |  |  |  |  |  |  |  |
|  |  |  |  |  |  |  |  |
| *DPPG / DPPTC* |  |  |  |  |  |  |  |
|  |  |  |  |  |  |  |  |
| blank | 25 | 3.47 | 4.17 | 2.11 | 0.423 | -0.205 | 37.4 |
| + SARS_FP_ | 25 | 2.75 | 3.55 | 2.59 | 0.442 | -0.231 | 35.7 |
| + SARS_IFP_ | 25 | 2.95 | 3.31 | 2.51 | 0.437 | -0.224 | 36.6 |
|  |  |  |  |  |  |  |  |
| blank | 37 | 3.47 | 4.47 | 2.04 | 0.490 | -0.216 | 35.8 |
| + SARS_FP_ | 37 | 2.84 | 3.57 | 2.52 | 0.467 | -0.202 | 34.9 |
| + SARS_IFP_ | 37 | 2.97 | 3.04 | 2.61 | 0.430 | -0.154 | 35.1 |
|  |  |  |  |  |  |  |  |
| blank | 45 | 4.04 | 3.77 | 1.96 | -0.367 | -0.064 | 33.9 |
| + SARS_FP_ | 45 | 2.84 | 3.57 | 2.05 | -0.333 | 0.046 | 33.9 |
| + SARS_IFP_ | 45 | 3.60 | 3.60 | 2.15 | -0.326 | 0.042 | 33.9 |
|  |  |  |  |  |  |  |  |
| *DPPG / 5-PCSL* |  |  |  |  |  |  |  |
|  |  |  |  |  |  |  |  |
| blank | 25 | 3.60 | 3.60 | 2.12 | 0.818 | - | 33.8 |
| + SARS_FP_ | 25 | 2.73 | 2.73 | 2.83 | 0.848 | - | 33.8 |
| + SARS_IFP_ | 25 | 3.52 | 3.52 | 2.21 | 0.838 | - | 33.8 |
|  |  |  |  |  |  |  |  |
| blank | 37 | 4.04 | 4.04 | 1.93 | 0.502 | - | 34.0 |
| + SARS_FP_ | 37 | 3.68 | 3.68 | 2.12 | 0.563 | - | 34.0 |
| + SARS_IFP_ | 37 | 3.95 | 3.95 | 1.94 | 0.548 | - | 34.0 |
|  |  |  |  |  |  |  |  |
| blank | 45 | 9.69 | 9.69 | 0.807 | 0.372 | - | 33.7 |
| + SARS_FP_ | 45 | 8.88 | 8.88 | 0.869 | 0.395 | - | 33.7 |
| + SARS_IFP_ | 45 | 9.03 | 9.03 | 0.856 | 0.385 | - | 33.7 |
|  |  |  |  |  |  |  |  |
| *DPPG / 16-PCSL* |  |  |  |  |  |  |  |
|  |  |  |  |  |  |  |  |
| blank | 25 | 7.53 | 7.53 | 1.03 | 0.334 | - 0.189 | 33.7 |
| + SARS_FP_ | 25 | 7.53 | 7.53 | 1.03 | 0.351 | - 0.172 | 33.7 |
| + SARS_IFP_ | 25 | 7.70 | 7.70 | 1.00 | 0.344 | -0.174 | 33.7 |
|  |  |  |  |  |  |  |  |
| blank |  |  |  |  |  |  |  |
| site 1 - 90% | 37 | 12.49 | 12.49 | 0.620 | 0.282 | - 0.086 | 33.7 |
| site 2 - 10% | 37 | 14.01 | 14.01 | 0.549 | 0.066 | - | 33.7 |
| + SARS_FP_ |  |  |  |  |  |  |  |
| site 1 - 76% | 37 | 12.49 | 12.49 | 0.620 | 0.327 | - 0.120 | 33.7 |
| site 2 - 24% | 37 | 12.21 | 12.21 | 0.639 | 0.092 | - | 33.7 |
| + SARS_IFP_ |  |  |  |  |  |  |  |
| site 1 - 75% | 37 | 12.49 | 12.49 | 0.547 | 0.303 | -0.084 | 33.7 |
| site 2 - 25% | 37 | 14.01 | 14.01 | 0.620 | 0.081 | - | 33.7 |
|  |  |  |  |  |  |  |  |
| blank | 45 | 38.6 | 38.6 | 0.198 | 0.039 | - 0.175 | 33.4 |
| + SARS_FP_ | 45 | 36.0 | 36.0 | 0.217 | 0.051 | - 0.144 | 33.5 |
| + SARS_IFP_ | 45 | 34.4 | 34.4 | 0.227 | 0.044 | - 0.139 | 33.6 |
|  |  |  |  |  |  |  |  |

**Table S4.** Best-fit rotational diffusion rates (R_┴_, R//), rotational correlation time (τ), order parameters (S_0_, S_2_), and A_zz_ component obtained from NLLS simulations of DPPTC, 5-PCSL, and 16-PCSL ESR spectra in DPPS model membranes at selected temperatures in the absence and in the presence of 5 mol% of peptides.

|  |  |  |  |  |  |  | |  | |
| --- | --- | --- | --- | --- | --- | --- | --- | --- | --- |
| System | T (^o^C) | R_┴_ (×10^7^ s^-1^) | R// (×10^8^ s^-1^) | τ (ns) | S_0_ | | S_2_ | | A_zz_ (G) |
|  |  |  |  |  |  | |  | |  |
|  |  |  |  |  |  | |  | |  |
| *DPPS / DPPTC* |  |  |  |  |  | |  | |  |
|  |  |  |  |  |  | |  | |  |
| blank | 37 | 5.71 | 3.07 | 1.66 | 0.255 | | - 0.099 | | 37.2 |
| + SARS_FP_ | 37 | 4.26 | 3.89 | 1.89 | 0.433 | | - 0.157 | | 38.2 |
| + SARS_IFP_ | 37 | 4.61 | 3.57 | 1.84 | 0.427 | | - 0.167 | | 38.2 |
|  |  |  |  |  |  | |  | |  |
| blank | 50 | 6.56 | 4.97 | 1.30 | 0.229 | | - 0.082 | | 36.5 |
| + SARS_FP_ | 50 | 5.37 | 3.98 | 1.59 | 0.381 | | - 0.047 | | 36.7 |
| + SARS_IFP_ | 50 | 5.67 | 4.40 | 1.47 | 0.363 | | - 0.034 | | 36.6 |
|  |  |  |  |  |  | |  | |  |
| blank | 60 | 4.90 | 8.91 | 1.31 | - 0.310 | | 0.101 | | 36.2 |
| + SARS_FP_ | 60 | 4.37 | 10.47 | 1.34 | - 0.282 | | 0.079 | | 36.2 |
| + SARS_IFP_ | 60 | 4.50 | 9.85 | 1.34 | - 0.292 | | 0.086 | | 36.2 |
|  |  |  |  |  |  | |  | |  |
| *DPPS / 5-PCSL* |  |  |  |  |  | |  | |  |
|  |  |  |  |  |  | |  | |  |
| blank | 60 | 14.7 | 14.7 | 0.528 | 0.304 | | - | | 35.0 |
| + SARS_FP_ | 60 | 13.4 | 13.4 | 0.574 | 0.316 | | - | | 35.1 |
| + SARS_IFP_ | 60 | 14.0 | 14.0 | 0.557 | 0.311 | | - | | 35.1 |
|  |  |  |  |  |  | |  | |  |
| *DPPS / 16-PCSL* |  |  |  |  |  | |  | |  |
|  |  |  |  |  |  | |  | |  |
| blank | 37 | 13.4 | 13.4 | 0.580 | 0.335 | | -0.202 | | 32.7 |
| + SARS_FP_ | 37 | 11.1 | 11.1 | 0.698 | 0.310 | | -0.171 | | 32.7 |
| + SARS_IFP_ | 37 | 12.7 | 12.7 | 0.610 | 0.320 | | -0.222 | | 32.7 |
|  |  |  |  |  |  | |  | |  |
| blank | 50 | 25.5 | 25.5 | 0.306 | 0.304 | | -0.190 | | 32.6 |
| + SARS_FP_ |  |  |  |  |  | |  | |  |
| site 1 - 75% | 50 | 25.5 | 25.5 | 0.306 | 0.304 | | -0.190 | | 33.6 |
| site 2 - 25% | 50 | 18.1 | 18.1 | 0.433 | 0.057 | | - | | 33.3 |
| + SARS_IFP_ |  |  |  |  |  | |  | |  |
| site 1 - 87.5% | 50 | 25.5 | 25.5 | 0.306 | 0.304 | | -0.190 | | 33.6 |
| site 2 - 12.5% | 50 | 25.5 | 25.5 | 0.306 | 0.058 | | - | | 34.0 |
|  |  |  |  |  |  | |  | |  |
| blank | 60 | 65.5 | 65.5 | 0.119 | 0.053 | | -0.189 | | 33.3 |
| + SARS_FP_ | 60 | 58.4 | 58.4 | 0.133 | 0.051 | | -0.158 | | 33.3 |
| + SARS_IFP_ | 60 | 61.2 | 61.2 | 0.127 | 0.053 | | - 0.169 | | 33.3 |
|  |  |  |  |  |  | |  | |  |

**Table S5.** Best-fit rotational diffusion rates (R_┴_, R//), rotational correlation time (τ), order parameters (S_0_, S_2_), and A_zz_ component obtained from NLLS simulations of DPPTC, 5-PCSL, and 16-PCSL ESR spectra in POPA model membranes at selected temperatures in the absence and in the presence of 5 mol% of peptides.

|  |  |  |  |  |  |  |  |
| --- | --- | --- | --- | --- | --- | --- | --- |
| System | T (^o^C) | R_┴_ (×10^7^ s^-1^) | R// (×10^8^ s^-1^) | τ (ns) | S_0_ | S_2_ | A_zz_ (G) |
|  |  |  |  |  |  |  |  |
|  |  |  |  |  |  |  |  |
| *POPA / DPPTC* |  |  |  |  |  |  |  |
|  |  |  |  |  |  |  |  |
| blank | 25 | 3.60 | 2.07 | 2.59 | - 0.299 | 0.156 | 37.5 |
| + SARS_FP_ | 25 | 3.89 | 2.04 | 2.45 | - 0.284 | 0.089 | 37.7 |
| + SARS_IFP_ | 25 | 4.14 | 2.22 | 2.31 | - 0.289 | 0.049 | 37.7 |
|  |  |  |  |  |  |  |  |
| blank | 37 | 4.71 | 3.66 | 1.77 | - 0.290 | 0.080 | 36.9 |
| + SARS_FP_ | 37 | 4.61 | 3.58 | 1.84 | - 0.290 | 0.080 | 36.9 |
| + SARS_IFP_ | 37 | 4.82 | 3.74 | 1.75 | - 0.290 | 0.080 | 36.9 |
|  |  |  |  |  |  |  |  |
| *POPA / 5-PCSL* |  |  |  |  |  |  |  |
|  |  |  |  |  |  |  |  |
| blank | 25 | 7.19 | 7.19 | 1.17 | 0.487 | - | 35.0 |
| + SARS_FP_ | 25 | 5.45 | 5.45 | 1.40 | 0.498 | - | 35.0 |
| + SARS_IFP_ | 25 | 6.12 | 6.12 | 1.27 | 0.499 | - | 35.0 |
|  |  |  |  |  |  |  |  |
| blank | 37 | 9.48 | 9.48 | 0.822 | 0.406 | - | 34.9 |
| + SARS_FP_ | 37 | 8.84 | 8.84 | 0.874 | 0.411 | - | 35.0 |
| + SARS_IFP_ | 37 | 9.05 | 9.05 | 0.854 | 0.412 | - | 35.2 |
|  |  |  |  |  |  |  |  |
| *POPA / 16-PCSL* |  |  |  |  |  |  |  |
|  |  |  |  |  |  |  |  |
| blank | 25 | 25.5 | 25.5 | 0.304 | 0.149 | - 0.301 | 32.7 |
|  |  |  |  |  |  |  |  |
| + SARS_FP_ |  |  |  |  |  |  |  |
| site 1 - 50% | 25 | 25.5 | 25.5 | 0.304 | 0.144 | - 0.277 | 32.2 |
| site 2 - 50% | 25 | 8.06 | 8.06 | 0.964 | 0.219 | - | 32.9 |
|  |  |  |  |  |  |  |  |
| + SARS_IFP_ |  |  |  |  |  |  |  |
| site 1 - 38% | 25 | 25.5 | 25.5 | 0.304 | 0.144 | - 0.277 | 32.2 |
| site 2 - 62% | 25 | 9.05 | 9.05 | 0.853 | 0.204 | - | 32.9 |
|  |  |  |  |  |  |  |  |
| blank | 37 | 29.3 | 29.3 | 0.263 | 0.100 | - 0.270 | 33.1 |
| + SARS_FP_ | 37 | 27.3 | 27.3 | 0.284 | 0.101 | - 0.273 | 33.1 |
| + SARS_IFP_ | 37 | 28.0 | 28.0 | 0.276 | 0.101 | - 0.273 | 33.1 |
|  |  |  |  |  |  |  |  |

**Table S6.** Best-fit rotational diffusion rates (R_┴_, R//), rotational correlation time (τ), order parameters (S_0_, S_2_), and A_zz_ component obtained from NLLS simulations of DPPTC in equimolar mixtures of DPPC/DPPG and DPPC/POPA model membranes at 37^o^C in the absence and in the presence of either 5 mol% of peptides or 10 mol% of linoleic acid (LA) or 16:0 lyso-PC (LPC).

|  |  |  |  |  |  |  |
| --- | --- | --- | --- | --- | --- | --- |
| System | R_┴_ (×10^7^ s^-1^) | R// (×10^8^ s^-1^) | τ (ns) | S_0_ | S_2_ | A_zz_ (G) |
|  |  |  |  |  |  |  |
|  |  |  |  |  |  |  |
| *DPPC / DPPG* |  |  |  |  |  |  |
|  |  |  |  |  |  |  |
| blank | 3.26 | 3.83 | 2.26 | 0.470 | - 0.203 | 35.0 |
| + SARS_FP_ | 2.53 | 3.66 | 2.71 | 0.493 | - 0.220 | 34.1 |
| + SARS_IFP_ | 2.57 | 3.31 | 2.73 | 0.486 | - 0.180 | 34.1 |
| + LA | 3.44 | 3.44 | 2.25 | 0.517 | - 0.212 | 35.0 |
| + LPC | 3.34 | 2.97 | 2.42 | 0.426 | - 0.186 | 35.0 |
|  |  |  |  |  |  |  |
| *DPPC / POPA* |  |  |  |  |  |  |
|  |  |  |  |  |  |  |
| blank | 3.83 | 2.59 | 2.32 | - 0.327 | 0.042 | 34.4 |
| + SARS_FP_ | 3.34 | 2.42 | 2.58 | - 0.238 | - 0.009 | 34.4 |
| + SARS_IFP_ | 3.66 | 2.47 | 2.43 | - 0.318 | 0.037 | 34.4 |
| + LA | 3.66 | 2.65 | 2.33 | - 0.310 | 0.034 | 34.4 |
| + LPC | 3.86 | 2.73 | 2.26 | - 0.335 | 0.058 | 34.4 |
|  |  |  |  |  |  |  |

**Table S7**. Summary of the empirical parameters defined over the amplitude FT spectra of n-PCSL (n = 0, 5, 7, 10, 12, and 16) in different model membranes. I(^2^H) corresponds to the deuterium intensity at 2.11 MHz and Δ represents de quadrupole intensity. Lipid model membranes: DPPC, POPC, POPC/cholesterol 7/3 (mol/mol), POPC/POPG 7/3 (mol/mol). In the latter, peptides SARS_FP_ and SAR_IFP_ were added at 1/20 peptide/lipid molar ratio. Standard deviations were calculated from duplicates.

|  |  |  |  |
| --- | --- | --- | --- |
| Sample | I (^2^H, 2.1 MHz)  (ns) | I (^2^H, 2.2 MHz)  (ns) | Δ  (ns) |
|  |  |  |  |
|  |  |  |  |
| POPC / POPG / DOPTC | 61.9 ± 1.2 | 40.6 ± 1.3 | 21.3 ± 0.5 |
| +SARS_FP_ | 65.9 ± 1.0 | 46.4 ± 1.2 | 19.5 ± 0.5 |
| +SARS_IFP_ | 64.1 ± 1.3 | 45.5 ± 1.2 | 18.6 ± 0.6 |
|  |  |  |  |
| POPC / POPG / 5-PCSL | 41.5 ± 1.0 | 26.9 ± 1.1 | 14.3 ± 0.7 |
| +SARS_FP_ | 35.4 ± 0.8 | 24.9 ± 0.6 | 10.5 ± 0.6 |
| +SARS_IFP_ | 33.1 ± 0.8 | 22.5 ± 0.7 | 10.6 ± 0.6 |
|  |  |  |  |
| POPC / POPG / 16-PCSL | 12.7 ± 0.6 | 9.3 ± 0.5 | 3.4 ± 0.8 |
| +SARS_FP_ | 10.0 ± 0.6 | 7.6 ± 0.6 | 1.3 ± 0.4 |
| +SARS_IFP_ | 9.8 ± 0.6 | 8.7 ± 0.5 | 1.1 ± 0.4 |
|  |  |  |  |
| POPC / Chol / DOPTC | 138.5 ± 2.1 | 105.0 ± 2.0 | 35.8 ± 1.0 |
| POPC / Chol / 5-PCSL | 69.9 ± 1.3 | 50.0 ± 1.2 | 19.9 ± 1.0 |
| POPC / Chol / 7-PCSL | 46.8 ± 1.4 | 32.9 ± 1.3 | 13.9 ± 0.7 |
| POPC / Chol / 10-PCSL | 2.3 ± 0.5 | 1.1 ± 0.4 | 1.2 ± 0.4 |
| POPC / Chol / 12-PCSL | 3.1 ± 0.5 | 2.3 ± 0.3 | 0.8 ± 0.3 |
| POPC / Chol / 16-PCSL | –^*^ | –^*^ | –^*^ |
|  |  |  |  |
| DPPC / DOPTC | 85.1 ± 2.2 | 60.0 ± 2.0 | 25.1 ± 1.0 |
| DPPC / 5-PCSL | 41.5 ± 1.1 | 29.3 ± 1.2 | 12.2 ± 0.9 |
| DPPC / 16-PCSL | 10.8 ± 1.1 | 8.6 ± 1.0 | 2.2 ± 0.6 |
|  |  |  |  |

^*^Deuterium signal not detected

**References**

1 Freed, J. *Theory of slow tumbling ESR spectra for nitroxides*. 53-130 (Academic Press, 1976).

2 Meirovitch, E., Nayeem, A. & Freed, J. Analysis of protein-lipid interactions based on model simulations of electron spin resonance spectra. *Journal of Physical Chemistry* **88**, 3454-3465 (1984).

3 Koller, D. & Lohner, K. The role of spontaneous lipid curvature in the interaction of interfacially active peptides with membranes. *Biochimica Et Biophysica Acta-Biomembranes* **1838**, 2250-2259, doi:10.1016/j.bbamem.2014.05.013 (2014).

4 Helfrich, W. Elastic properties of lipid bilayers - theory and possible experiments. *Zeitschrift Fur Naturforschung C-a Journal of Biosciences* **C 28**, 693-703 (1973).

5 Helfrich, W. *Amphiphilic mesophases made of defects*. 716–755 (North-Holland Publishing, 1981).

6 Ben-Shaul, A. *Molecular theory of lipid-chain packing, elasticity and lipid-protein interaction in lipid bilayers*. 359–401 (Elsevier/North Holland, 1995).

7 Brown, M. Curvature Forces in Membrane Lipid-Protein Interactions. *Biochemistry* **51**, 9782-9795, doi:10.1021/bi301332v (2012).

8 Ge, M. & Freed, J. Fusion Peptide from Influenza Hemagglutinin Increases Membrane Surface Order: An Electron-Spin Resonance Study. *Biophysical Journal* **96**, 4925-4934, doi:10.1016/j.bpj.2009.04.015 (2009).

9 Lai, A. & Freed, J. HIV gp41 Fusion Peptide Increases Membrane Ordering in a Cholesterol-Dependent Fashion. *Biophysical Journal* **106**, 172-181, doi:10.1016/j.bpj.2013.11.027 (2014).
